# On-treatment viral factors affect subsequent hepatitis B surface antigen seroclearance in patients treated with nucleos(t)ide analogs for > 10 years

**DOI:** 10.1007/s00535-026-02415-3

**Published:** 2026-04-21

**Authors:** Tetsuya Hosaka, Hayato Hikita, Yuki Tahata, Ryoko Yamada, Kazuhiro Murai, Masanori Miyazaki, Hisashi Ishida, Atsushi Hosui, Ryotaro Sakamori, Nobuyuki Tatsumi, Yoshinori Doi, Kazuyoshi Ohkawa, Satoshi Egawa, Takatoshi Nawa, Yasutoshi Nozaki, Kazuho Imanaka, Masanori Nakahara, Mitsuru Sakakibara, Takayuki Yakushijin, Yuichi Yoshida, Hiroyuki Ogawa, Takeo Usui, Kengo Matsumoto, Kazuki Maesaka, Kumiko Shirai, Yuki Makino, Yoshinobu Saito, Takahiro Kodama, Tetsuo Takehara

**Affiliations:** 1 Department of Gastroenterology and Hepatology, The University of Osaka Graduate School of Medicine, Suita, Japan; 2 Department of Hepatology, Toranomon Hospital, Tokyo, Japan; 3 Department of Gastroenterology, Osaka Keisatsu Hospital, Osaka, Japan; 4 Department of Gastroenterology and Hepatology, Ikeda Municipal Hospital, Ikeda, Japan; 5 Department of Gastroenterology and Hepatology, Osaka Rosai Hospital, Sakai, Japan; 6 Department of Gastroenterology and Hepatology, NHO Osaka National Hospital, Osaka, Japan; 7 Department of Gastroenterology and Hepatology, Japan Community Healthcare Organization Osaka Hospital, Osaka, Japan; 8 Department of Gastroenterology and Hepatology, Otemae Hospital, Osaka, Japan; 9 Department of Hepatobiliary and Pancreatic Oncology, Osaka International Cancer Institute, Osaka, Japan; 10 Department of Gastroenterology and Hepatology, Kinki Central Hospital of Mutual Aid Association of Public School Teachers, Itami, Japan; 11 Department of Gastroenterology and Hepatology, Higashiosaka City Medical Center, Osaka, Japan; 12 Department of Gastroenterology and Hepatology, Kansai Rosai Hospital, Amagasaki, Japan; 13 Department of Gastroenterology and Hepatology, Itami City Hospital, Itami, Japan; 14 Department of Gastroenterology and Hepatology, Minoh City Hospital, Minoh, Japan; 15 Department of Gastroenterology and Hepatology, Yao Municipal Hospital, Yao, Japan; 16 Department of Gastroenterology and Hepatology, Osaka General Medical Center, Osaka, Japan; 17 Department of Gastroenterology and Hepatology, Suita Municipal Hospital, Suita, Japan; 18 Department of Gastroenterology, Nishinomiya Municipal Central Hospital, Nishinomiya, Japan; 19 Department of Gastroenterology and Hepatology, Ashiya Municipal Hospital, Ashiya, Japan; 20 Department of Gastroenterology and Hepatology, Toyonaka Municipal Hospital, Toyonaka, Japan

**Keywords:** HBsAg seroclearance, HBeAg, Functional cure, Nucleos(t)ide analogs

## Abstract

**Background:**

Long-term nucleos(t)ide analog (NUC) treatment improves the outcomes of patients with chronic hepatitis B virus (HBV) infection. However, only a limited number of patients treated with NUC can achieve hepatitis B surface antigen (HBsAg) seroclearance, the so-called “functional cure.” However, it remains unclear how on-treatment viral factors affect HBsAg seroclearance during long-term NUC treatment. We aimed to investigate whether the baseline and on-treatment HBV markers can predict HBsAg seroclearance and reduction in patients treated with long-term NUC treatment.

**Methods:**

This study included two independent cohorts consisting of 843 patients in the derivation cohort and 1781 patients in the validation cohort.

**Results:**

HBsAg seroclearance was infrequent (3.7–6.2%), with annual rates of 4–10/1,000 person-years in the derivation and validation cohorts. In baseline hepatitis B e-antigen (HBeAg)-positive patients, early on-treatment HBeAg loss strongly predicted subsequent HBsAg seroclearance and a reduction of < 10 IU/mL, whereas delayed or absent HBeAg loss rarely followed HBsAg seroclearance. A simple predictive model for HBsAg seroclearance based on earlier HBeAg loss, sex, and age was developed (SLOPES50). In baseline HBeAg-negative patients, low baseline or on-treatment HBsAg levels (< 100 IU/mL) were key predictors of HBsAg seroclearance or a reduction of < 10 IU/mL. Landmark analysis and time-dependent Cox regression analyses confirmed these associations in both cohorts. A substantial number of patients remained HBsAg ≥ 100 even after 15 years of NUC treatment in both cohorts.

**Conclusions:**

Functional cure during prolonged NUC treatment of > 10 years depends on early virological responses in both HBeAg-positive and HBeAg-negative patients.

**Supplementary Information:**

The online version contains supplementary material available at 10.1007/s00535-026-02415-3.

## Introduction

More than 254 million people worldwide are persistently infected with hepatitis B, with 1.1 million chronic hepatitis B (CHB) progressing to cirrhosis and hepatocellular carcinoma (HCC), resulting in death. [1] More than 20 years have passed since the treatment of patients with CHB using nucleos(t)ide analogs (NUCs) became available. Long-term treatment with NUCs reduces hepatitis B virus (HBV) DNA levels and thus decreases the risk of HCC development, fibrosis progression, and liver-related death in patients with CHB. [2–6] Although long-term NUC treatment contributes to an improved prognosis, the ultimate goal of HBV treatment is a functional cure, defined as hepatitis B surface antigen (HBsAg) seroclearance. Some guidelines suggest prolonging NUC treatment until HBsAg seroclearance is achieved. [7–9] NUC compounds do not directly lower HBsAg levels. [10] Because only a small number of patients can achieve HBsAg seroclearance even with long-term NUC treatment, several patients are forced to receive nearly lifelong treatment. [11, 12] In Japan, no insurance coverage limitation exists for NUC treatment duration. [13] Therefore, currently, a large number of patients who have been treated with NUC for > 10 years exists, and how to manage these patients with lifelong NUC treatment is an issue. To achieve this, evaluating the status and factors associated with HBsAg seroclearance in patients receiving long-term NUC treatment is necessary.

The baseline factors associated with HBsAg seroclearance in long-term NUC treatment include HBV genotype, ethnicity, high baseline alanine aminotransferase (ALT) levels, and low HBsAg levels. [11, 12] HBsAg decline in the first 6 months is associated with subsequent HBsAg seroclearance during long-term NUC treatment. [14] However, the association between long-term on-treatment factors during NUC treatment and subsequent HBsAg seroclearance remains unclear. Recently, novel HBV therapies with various mechanisms of action have been developed to improve HBsAg seroclearance. Several clinical trials are being conducted to identify novel agents for patients receiving NUC treatment. [10, 15] New agents that inhibit HBV RNA tend to reduce the large amount of HBsAg levels, particularly in patients with low HBsAg levels [16, 17]. Therefore, evaluating the actual HBsAg trends in long-term NUC-treated patients to increase the number of patients who achieve HBsAg seroclearance with upcoming new therapies is important.

This study aimed to clarify the actual status of HBsAg seroclearance or decline in long-term NUC treatment > 10 years, identify the optimal targets in patients with CHB for new therapies for HBV that may develop in the future, and estimate the actual treatment burden in patients treated with long-term NUC.

## Materials and methods

### Patients and study design

We conducted a multicenter retrospective cohort study of patients with CHB who received long-term NUC treatment using two independent, large-scale cohorts. We consecutively recruited 843 patients treated with NUCs, including 154 patients initially treated with lamivudine, 618 with entecavir (ETV), 46 with tenofovir disoproxil fumarate (TDF), and 25 with tenofovir alafenamide fumarate (TAF), as the derivation cohort, named the Osaka Liver Forum, which consisted of 19 centers located in Western Japan (Supplementary Fig. 1). Treatment criteria were based on the Japanese Society of Hepatology guidelines for the management of HBV infection. [9] The validation cohort consisted of 1781 patients who were enrolled using the same inclusion criteria as mentioned above from Toranomon Hospital in Tokyo, located in Eastern Japan. The details of the inclusion criteria for this study are provided in the Supplementary Information.

Informed consent was obtained by informing each patient of the public announcement of this study via a web page and bulletin boards in each institute because of the retrospective observational nature of this study. The study protocol complied with the ethical guidelines of the Declaration of Helsinki. The ethical guidelines for medical and health research involving human subjects of the Ministry of Health, Labour and Welfare in Japan were approved by the University of Osaka (ID: 12,380) and Ethics Committee of Toranomon Hospital (ID: 1530).

### Treatment monitoring and clinical data collection

All patients received continuous NUC treatment until HBsAg seroclearance was achieved. Some patients received a highly potent NUC agent, including ETV, TDF, or TAF, and switched from the first-line drug to prevent the emergence of drug resistance, based on each physician’s decision. Patients who experienced an HBV DNA relapse of ≥ 1 log copies from the nadir received rescue therapy with adefovir dipivoxil (ADV), TDF, or TAF. Additionally, some patients received TAF-containing regimens after switching from long-term TDF- or ADV-containing regimens because of renal impairment, decreased bone mineral density, or prevention by each physician. Patients were followed up until HBsAg seroclearance was confirmed, as described below, or until the last follow-up (before April 2020).

### Hepatitis B virus markers and study outcomes

HBsAg levels and HBV DNA were measured using commercially available assay kits. The details of each assay are provided in the Supplementary Information. Patients were followed up until HBsAg seroclearance was defined as achieving < 0.05 IU/mL (primary outcome) or until the last follow-up (before April 2020). Moreover, achieving HBsAg < 10 IU/mL was set as the secondary outcome of this study. This is because some clinical trials on new antiviral agents have applied endpoints, such as the NUC stopping criteria [17].

### Statistical analyses

Categorical data were compared using the chi-squared or Fisher’s exact test. Continuous variables with a nonparametric distribution were analyzed using the Mann–Whitney *U* test, whereas those with a parametric distribution were analyzed using Student’s *t-*test. Patients with the following events were censored: loss to follow-up or death prior to HBsAg seroclearance. The cumulative rates of HBsAg seroclearance or HBsAg < 10 IU/mL were analyzed using the Kaplan–Meier method, and differences in the resulting curves were evaluated using log-rank tests. Cox regression analyses were performed to assess the variables that were significantly associated with HBsAg seroclearance or HBsAg < 10 IU/mL. A multivariate Cox proportional hazards regression was used to calculate hazard ratios and 95% confidence intervals (95% CIs) for HBsAg seroclearance or achieving HBsAg < 10 IU/mL after controlling simultaneously for potential predictors of those outcomes. The multivariate models included variables that exhibited significant associations (P < 0.05) with HBsAg seroclearance or HBsAg < 10 IU/mL in a univariate analysis. The trends between the timing of HBeAg loss and HBsAg seroclearance or achievement of HBsAg < 10 IU/mL were evaluated using the Mantel–Cox test. Data analyses were performed using the SPSS software version 25.0 (IBM Corp., Armonk, NY, USA) and STATA version 17 (STATA Corp., USA).

## Results

### Patient characteristics

The baseline characteristics of the patients in the derivation cohort are shown in Table 1. The HBeAg + and HBeAg– cohorts included 386 and 457 patients, respectively (Table 1). During respective median follow-up durations of 10.5 and 9.6 years, 23 (6.0%) patients in the HBeAg + cohort (6.1/1,000 person-years) and 17 (3.7%) patients in the HBeAg– cohort achieved HBsAg seroclearance (4.2/1,000 person-years). The baseline characteristics of the patients in the validation cohort are shown in Supplementary Information. The median age was higher in the derivation cohort than in the validation cohort (52 vs. 44 years). The annual HBsAg seroclearance rate in the derivation cohort was similar to that in the validation cohort in patients with baseline HBeAg positivity but was lower in the derivation cohort in patients with baseline HBeAg negativity.

**Table 1:** Patients’ baseline characteristics according to hepatitis B e-antigen (HBeAg) status in the derivation cohort

*Baseline characteristics*	HBeAg positive (*N* = 386)	HBeAg negative (*N* = 457)	All (*N* = 843)	Missing, no (%)
Age (y)	48 (39–59)	54 (46–62)	52 (43–61)	3 (0.4%)
Sex (male), no. (%)	245 (63.5%)	258 (56.5%)	503 (59.7%)	0
Cirrhosis, no. (%)	79 (20.5%)	85 (18.6%)	164 (19.5%)	3 (0.4%)
HBeAg-positive, no. (%)	–	–	386 (45.8%)	0
HBV DNA (log copies/mL)	7.6 (6.8–8.3)	6.0 (4.9–6.9)	6.7 (5.5–7.6)	0
HBsAg > 250 IU/mL, no. (%)	341 (88.3%)	369 (80.7%)	710 (84.2%)	62 (7.4%)
ALT level (IU/L)	81 (50–173)	65 (37–138)	70 (43–158)	1 (0.1%)
GGTP level (IU/L)	49 (31–88)	45 (26–87)	31 (22–64)	38 (4.5%)
Serum albumin (g/L)	3.9 (3.6–4.1)	4.1 (3.8–4.4)	4.0 (3.7–4.3)	18 (2.1%)
Platelet (10^5^/mm^3^)	15.5 (11.4–19.1)	16.3 (12.1–19.8)	15.8 (11.9–19.6)	8 (0.9%)
Prior history of IFN Tx, no. (%)	61 (15.8%)	45 (9.9%)	106 (12.5%)	1 (0.1%)
First-line NUC (ETV:LAM:TAF:TDF)	275:88:16:7	343:66:30:18	618:154:46:25	0
Treatment duration (*y*)	10.5 (7.1–13.2)	9.6 (5.6–11.9)	10.0 (6.4–12.6)	
HBsAg seroclearance cases, no	23 (6.1/1000 *P*-*Y*)	17 (4.2/1000 *P*-*Y*)	40 (5.0/1000 *P*-*Y*)	

All values are expressed as medians (25th–75th percentiles) or numbers (percentages of total). *HBV* hepatitis B virus, *HBsAg* hepatitis B surface antigen, *ALT* alanine aminotransferase, *GGTP* amma-glutamyltransferase, *IFN* interferon, *NUC* nucleos(t)ide analog, *ETV* entecavir, *LAM* lamivudine, *TAF* tenofovir alafenamide, *TDF* tenofovir disoproxil fumarate

### Hepatitis B surface antigen (HBsAg) seroclearance or achieving HBsAg < 10 IU/mL after starting nucleos(t)ide analog (NUC) treatment according to hepatitis B e-antigen (HBeAg) status

Supplemental Fig. 3 shows the Kaplan–Meier curves representing the cumulative rates of HBsAg seroclearance or HBsAg < 10 IU/mL according to baseline HBeAg status in the derivation cohort. No significant differences were noted in HBsAg seroclearance or reduction in the baseline HBeAg status. The cumulative HBsAg seroclearance rates were 5.9% at year 10 and 7.5% at year 15 in patients with baseline HBeAg positivity and 5.5% at year 10 and 5.5% at year 15 in patients with baseline HBeAg negativity (Supplementary Fig. 3a). The cumulative rates of HBsAg < 10 IU/mL were 9.5% at year 10 and 21.5% at year 15 in patients with baseline HBeAg positivity and 11.0% at year 10 and 24.0% at year 15 in patients with baseline HBeAg negativity (Supplementary Fig. 3b).

### Factors associated with HBsAg seroclearance or HBsAg < 10 IU/mL

Table 2 shows the baseline and on-treatment factors associated with HBsAg seroclearance according to the HBeAg status, as identified in the univariate and multivariate analyses using time-dependent Cox regression in the derivation cohort. In the HBeAg-positive cohort, multivariate analysis showed that on-treatment HBeAg loss, a time-dependent variable, was associated with HBsAg seroclearance after adjustment (Table 2). In contrast, low baseline HBsAg levels were only associated with HBsAg seroclearance in a univariate analysis of the HBeAg-negative cohort (Table 2). Supplementary Table 2a shows that on-treatment HBeAg loss was associated with HBsAg seroclearance in baseline HBeAg-positive patients in the validation cohort. Supplementary Table 4a shows that low baseline HBsAg levels were associated with HBsAg seroclearance in the baseline HBeAg-negative validation cohort.

**Table 2:** Factors associated with HBsAg seroclearance according to baseline HBeAg status in the time-dependent model

Baseline HBeAg-positive				
Variable	Univariate HR (95% CI)	*P*	Multivariate-adjusted HR (95% CI)^†^	*P*
Age (y)	1.06 (1.02–1.09)	0.001	1.06 (1.03–1.10)	< 0.001
Sex (male), no. (%)	3.91 (1.16–13.17)	0.028	5.59 (1.49–20.8)	0.011
Cirrhosis, no. (%)	0.58 (0.17–1.95)	0.378		
HBV DNA (log copies/mL)	0.91 (0.63–1.30)	0.593		
HBsAg > 250 IU/mL	0.32 (0.07–1.44)	0.138		
ALT level (IU/L)	1.03 (0.98–1.07)	0.216		
GGTP level (IU/L)	0.999 (0.993–1.005)	0.742		
Serum albumin (g/L)	0.57 (0.28–1.18)	0.130		
Platelet (105/mm^3^)	1.08 (1.02–1.14)	0.009	1.12 (1.05–1.20)	0.001
History of IFN Tx	1.41 (0.52–3.83)	0.499		
First-line NUC				
LAM	1	Ref		
ETV	0.78 (0.31–1.13)	0.596		
TDF or TAF	2.27 (0.26–19.88)	0.460		
On-treatment HBeAg loss (time-dependent covariate)	63.6 (8.13–497.9)	< 0.001	54.0 (6.83–426.0)	< 0.001

Baseline HBeAg-negative				
Variable	Univariate HR (95% CI)	*P*	Multivariate-adjusted HR (95% CI)^†^	*P*
Age (y)	0.96 (0.92–1.01)	0.106		
Sex (male), no. (%)	1.04 (0.40–2.74)	0.931		
Cirrhosis, no. (%)	0.26 (0.03–1.96)	0.190		
HBV DNA (log copies/mL)	0.97 (0.69–1.38)	0.882		
HBsAg > 250 IU/mL	0.10 (0.03–0.27)	< 0.001		
ALT level (IU/L)	1.02 (0.97–1.07)	0.519		
GGTP level (IU/L)	1.002 (0.998–1.005)	0.371		
Serum albumin (g/L)	1.66 (0.54–5.17)	0.379		
Platelet (105/mm^3^)	1.01 (0.933–1.09)	0.872		
History of IFN Tx	1.06 (0.24–4.64)	0.938		
First-line NUC				
LAM	1	Ref		
ETV	1.81 (0.41–7.95)	0.431		
TDF or TAF	NA^#^	NA^#^		

^†^Adjusted for age, sex, platelet counts, and on-treatment HBeAg loss

^#^Due to no event

Next, we evaluated the factors associated with achieving HBsAg < 10 IU/mL, which was the other endpoint. Supplementary Table 3 shows the baseline and on-treatment factors associated with achieving HBsAg < 10 IU/mL according to the HBeAg status, as identified in the univariate and multivariate analyses using time-dependent Cox regression in the derivation cohort. These results align with those obtained for HBsAg seroclearance. On-treatment HBeAg loss, a time-dependent variable, was associated with the subsequent achievement of HBsAg < 10 IU/mL in the HBeAg-positive derivation cohort (Supplementary Table 3a). In the HBeAg-negative cohort, low baseline HBsAg levels were only associated with HBsAg < 10 IU/mL and HBsAg seroclearance (Supplementary Table 3b). In the validation cohort, on-treatment HBeAg loss was associated with HBsAg < 10 IU/mL in HBeAg-positive patients (Supplementary Table 2b), and low baseline HBsAg levels were associated with HBsAg < 10 IU/mL in HBeAg-negative patients (Supplementary Table 4b). Altogether, these factors were associated with HBsAg seroclearance and HBsAg < 10 IU/mL.

### Association between on-treatment HBeAg loss and HBsAg seroclearance or achieving HBsAg < 10 IU/mL in patients with baseline HBeAg positivity

**Fig. 1:**
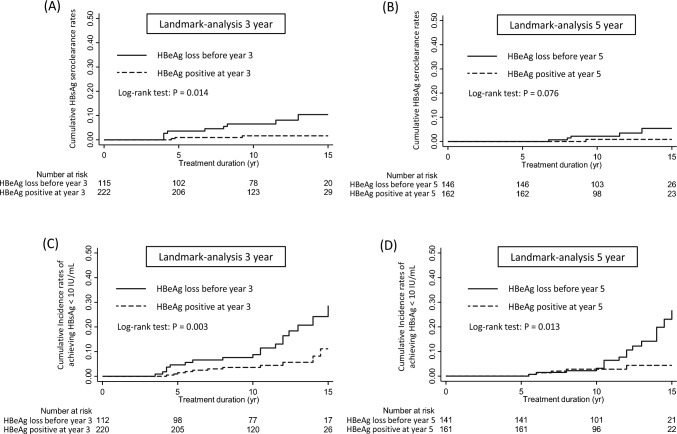
Cumulative incidence rates of HBsAg seroclearance or achieving HBsAg < 10 IU/mL using the landmark analysis among patients with baseline HBeAg positivity in the derivation cohort. **a** HBsAg seroclearance according to HBeAg status at year 3. The log-rank test revealed a statistically significant difference in the HBsAg seroclearance between patients with HBeAg loss before year 3 and without HBeAg loss (log-rank test: *P* < 0.001). **B** HBsAg seroclearance according to HBeAg status at year 5. The log-rank test revealed a statistically significant difference in the HBsAg seroclearance between patients with HBeAg loss before year 5 and without HBeAg loss (log-rank test: *P* < 0.001). **c** Achieving HBsAg < 10 IU/mL according to HBeAg status at year 3. The log-rank test revealed a statistically significant difference in the achievement of HBsAg < 10 IU/mL between patients with HBeAg loss before year 3 and without HBeAg loss (log-rank test: *P* < 0.001). **d** Achieving HBsAg < 10 IU/mL according to HBeAg status at year 5. The log-rank test revealed a statistically significant difference in the achievement of HBsAg < 10 IU/mL between patients with HBeAg loss before year 5 and without HBeAg loss (log-rank test: *P* < 0.001). *HBeAg* Hepatitis B e antigen, *HBsAg* Hepatitis B surface antigen

We performed a landmark analysis to evaluate the effects of on-treatment HBeAg loss on HBsAg seroclearance or HBsAg < 10 IU/mL in the baseline HBeAg-positive cohort. Figure 1 shows the cumulative incidence rates of HBsAg seroclearance (Fig. 1a and 1b) and HBsAg < 10 IU/mL (Fig. 1c and 1 d) in patients with baseline HBeAg positivity in the derivation cohort. HBsAg seroclearance rates were significantly higher in patients who achieved HBeAg loss before year 3 (Fig. 1a). A marginal difference was observed in HBsAg seroclearance between the patients with and without HBeAg loss (Fig. 1b). The cumulative incidence rates were higher in patients who achieved HBeAg loss before year 3 or 5 (Fig. 1c and d). In the landmark analysis of the validation cohort, HBsAg seroclearance or reduction was likely to occur in patients with early HBeAg loss and in the derivation cohort (Supplementary Fig. 4a–d).

Multivariate analysis showed that HBeAg loss before year 3 was significantly associated with HBsAg seroclearance; however, HBeAg loss before year 5 was not statistically significant owing to the small number of events (Table 3). In contrast, HBeAg loss before year 3 or 5 was significantly associated with HBsAg < 10 IU/mL (Supplementary Table 5a). The other details of this part are provided in the Supplementary Information.

**Table 3:** Association between on-treatment viral factors and HBsAg seroclearance in the baseline HBeAg-positive and -negative cohort (landmark analysis)

Baseline HBeAg-positive							
Factors		Total number	Cases	*PY*	Annual incidences/1000 PY (95% CI)	Adjusted HR (95% CI)^†^	*P*
HBeAg loss before year 3	No	222	4	2371.3	1.69 (0.63–4.49)	1	–
HBeAg loss before year 3	Yes	115	9	1317.9	6.83 (3.55–13.1)	3.64 (1.12–11.9)	0.032
HBeAg loss before year 5	No	162	1	1816.5	0.55 (0.08–3.91)	1	–
HBeAg loss before year 5	Yes	146	6	1750.3	3.43 (0.15–7.63)	5.28 (0.63–44.5)	0.124

Baseline HBeAg-negative							
Factors		Total number	Cases	PY	Annual incidences/1000 PY (95% CI)	Adjusted HR (95% CI) ^‡^	*P*
HBsAg < 100 IU/mL at year 3	No	327	4	3417.3	1.17 (0.44–3.12)	1	–
	Yes	44	9	337.5	23.8 (1.24–45.8)	76.6 (15.2–385.2)	< 0.001
HBsAg < 100 IU/mL at year 5	No	287	1	3200.4	0.3 (0.04–2.22)	1	–
	Yes	46	10	437.8	22.8 (12.3–42.5)	119.9 (13.5–1066)	< 0.001

^†^Adjusted for age and HBeAg loss

^‡^Adjusted for baseline and on-treatment HBsAg

### Association between on-treatment HBsAg levels and HBsAg seroclearance or achieving HBsAg < 10 IU/mL in patients with baseline HBeAg negativity

**Fig. 2:**
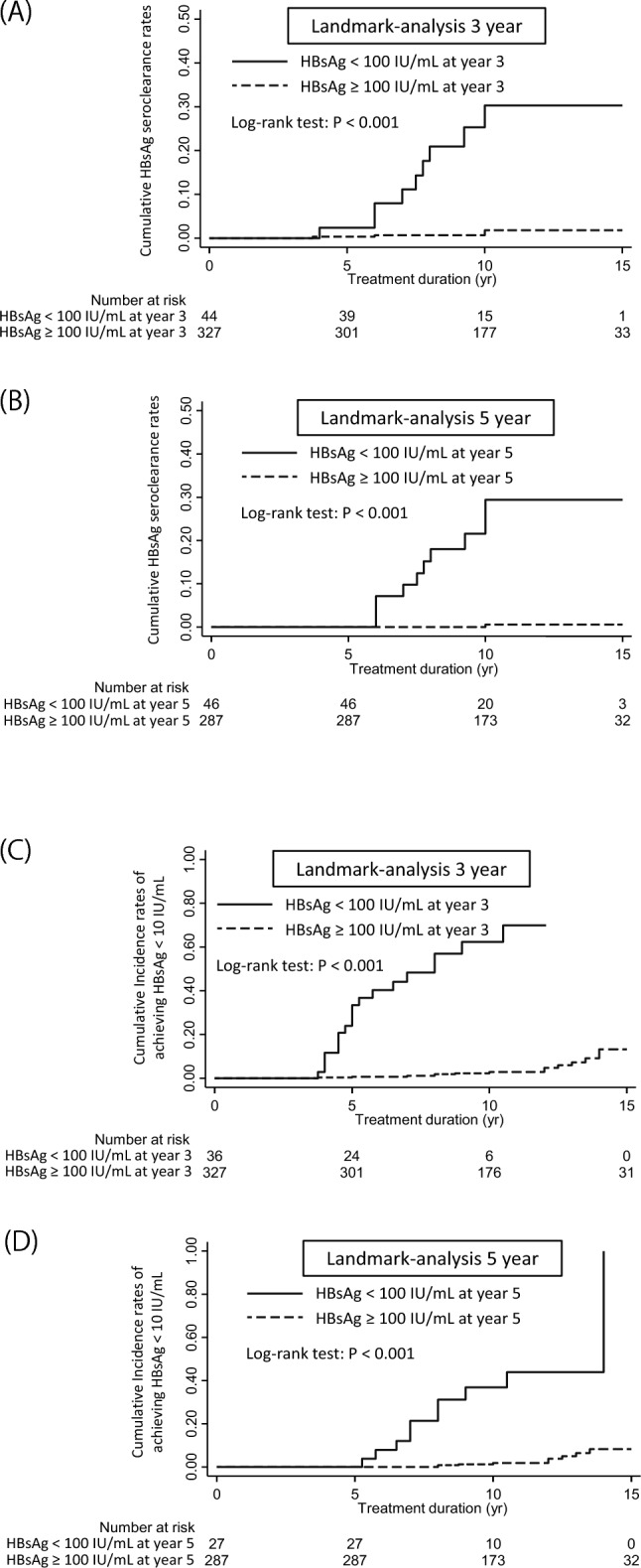
Cumulative incidence rates of HBsAg seroclearance or achieving HBsAg < 10 IU/mL using the landmark analysis among patients with baseline HBeAg negativity in the derivation cohort. **a** HBsAg seroclearance according to HBsAg 100 IU/mL at year 3. The log-rank test revealed a statistically significant difference in the HBsAg seroclearance between patients with HBsAg < 100 IU/mL at year 3 and without it (log-rank test: *P* < 0.001). **b** HBsAg seroclearance according to HBsAg level of 100 IU/mL at year 5. The log-rank test revealed a statistically significant difference in the HBsAg seroclearance between patients with HBsAg < 100 IU/mL at year 5 and without it (log-rank test: *P* < 0.001). **c** Achieving HBsAg < 10 IU/mL according to HBsAg level of 100 IU/mL at year 3. The log-rank test revealed a statistically significant difference in the achievement of HBsAg < 10 IU/mL between patients with HBsAg < 100 IU/mL at year 3 and without it (log-rank test: *P* < 0.001). **d** Achieving HBsAg < 10 IU/mL according to HBsAg level of 100 IU/mL at year 5. The log-rank test revealed a statistically significant difference in the achievement of HBsAg < 10 IU/mL between patients with HBsAg < 100 IU/mL at year 5 and without it (log-rank test: *P* < 0.001). *HBsAg* hepatitis B surface antigen

We performed a landmark analysis to evaluate the effects of on-treatment HBsAg levels on HBsAg seroclearance or HBsAg < 10 IU/mL in the baseline HBeAg-positive cohort. Figure 2 shows the cumulative incidence rates of HBsAg seroclearance (Fig. 2a and b) and HBsAg < 10 IU/mL (Fig. 2c and d). The cumulative incidence rates of HBsAg seroclearance and HBsAg < 10 IU/mL were significantly higher in patients with HBsAg < 100 IU/mL at year 3 (Fig. 2a and c). In the landmark analysis at year 5, the results were similar. The cumulative incidence rates of HBsAg seroclearance and HBsAg < 10 IU/mL were significantly higher in patients with HBsAg < 100 IU/mL at year 3 (Fig. 2b and d).

Multivariate analysis showed that HBsAg < 100 IU/mL at year 3 or 5 was significantly associated with both HBsAg seroclearance and HBsAg < 10 IU/mL after adjusting for baseline and HBsAg levels (Table 3, Supplementary Table 5b). The other details of this part are provided in the Supplementary Information.

### Likelihood of HBsAg seroclearance or HBsAg reduction after earlier HBeAg loss

**Fig. 3:**
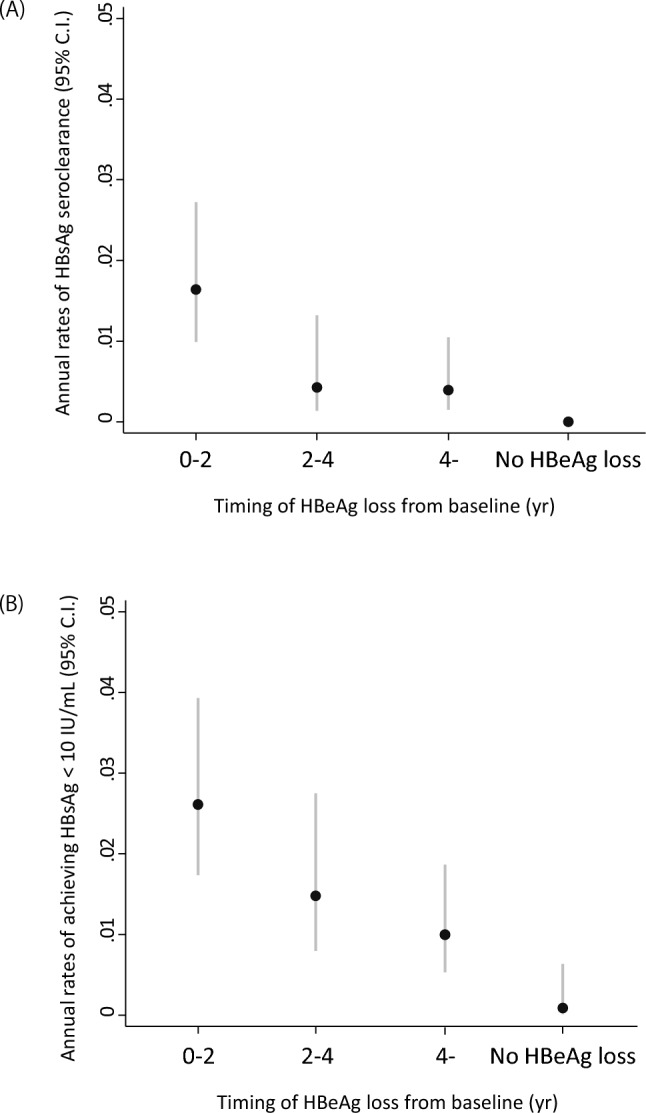
Annual rates of HBsAg seroclearance or achieving HBsAg < 10 IU/mL among patients with baseline HBeAg positivity in the derivation cohort according to the timing of HBeAg loss. **a** Annual rates of HBsAg seroclearance. The Mantel–Cox test revealed a statistically significant downtrend in the HBsAg seroclearance according to the delayed timing of HBeAg loss (P for trend < 0.001). **b** Annual rates of achieving HBsAg < 10 IU/mL. The Mantel–Cox test revealed a statistically significant downtrend in the achievement of HBsAg < 10 IU/mL according to the delayed timing of HBeAg loss (P for trend < 0.001). *HBsAg* hepatitis B surface antigen

On-treatment HBeAg loss occurred in 238 (61.7%) patients among baseline HBeAg-positive patients in the derivation cohort. The cumulative HBeAg loss rates were 49.5% at year 5, 69.8% at year 10, and 76.9% at year 15. Next, we evaluated whether the timing of HBeAg loss influenced the annual rates of HBsAg seroclearance or the achievement of HBsAg < 10 IU/mL in patients with baseline HBeAg positivity. We categorized these patients into four groups based on the timing of HBeAg loss: 0–2 years from baseline, 2–4 years, > 4 years, and no HBeAg loss. The annual rates of HBsAg seroclearance and HBsAg < 10 IU/mL in the derivation cohort are shown in Fig. 3a and b. Thus, a significant reverse trend was observed between the annual rates of HBsAg seroclearance or reduction and the timing of HBeAg loss (Mantel–Cox trend test, P < 0.001). Similar findings were observed in the validation cohort (Supplementary Fig. 6). Patients with delayed or no HBeAg loss had almost no chance of subsequent HBsAg seroclearance or achieving HBsAg < 10 IU/mL, even if they received prolonged NUC treatment in the derivation and validation cohorts with baseline HBeAg positivity. Moreover, we analyzed the factors associated with early HBeAg loss before year 3 among patients with baseline HBeAg positivity in both the derivation and validation cohorts (Supplementary Table 8). In both cohorts, low baseline HBV DNA levels were associated with early HBeAg loss, whereas sex was not.

**Fig. 4:**
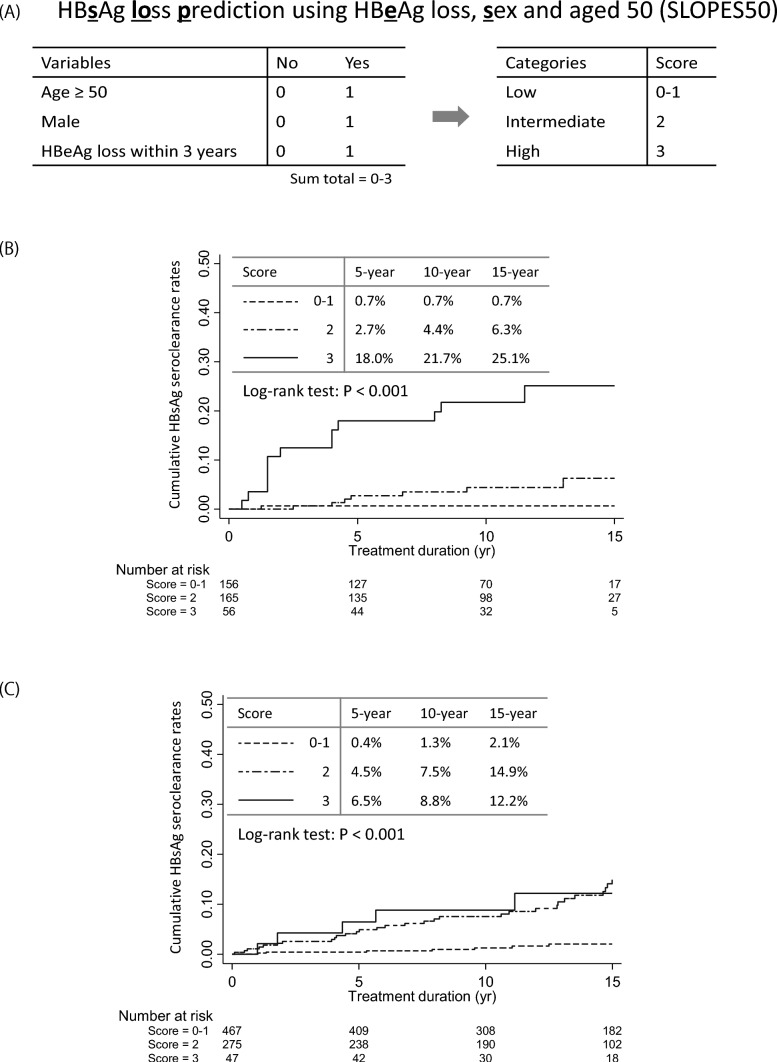
Simple prediction model of HBsAg seroclearance using HBeAg loss within 3 years, sex, and baseline age > 50 years in baseline HBeAg-positive patients (SLOPES50 score: HBsAg loss prediction using HBeAg loss, sex, and aged 50). **a** Calculating a score. **b** Cumulative incidence rates of HBsAg seroclearance stratified according to “SLOPES50” score in the derivation cohort. **c** Cumulative incidence rates of HBsAg seroclearance stratified according to “SLOPES50” score in the validation cohort. *HBeAg* hepatitis B e antigen, *HBsAg* hepatitis B surface antigen

Finally, we created a simple prediction model of HBsAg seroclearance using HBeAg loss within 3 years, sex, and baseline age because multivariate analysis showed that earlier HBeAg loss, sex, and baseline age were associated with HBsAg seroclearance in the derivation with baseline HBeAg positivity (SLOPES50 score: HBsAg loss prediction using HBeAg loss, sex, and age 50). We assigned 1 point to each patient if he or she was aged > 50 years at the start of NUC, if he was male, and if he or she achieved HBeAg loss within the first 3 years (Fig. 4a). The cutoff for baseline age was set at 50 years, rounded from the median age. The distribution of this score was 0–3, if a score was added. Figure 4 shows the cumulative incidence rates of HBsAg seroclearance stratified according to “SLOPES50” score in both the derivation and validation cohorts. The HBsAg seroclearance rate was stratified according to SLOPES score in the derivation cohort (Fig. 4b). In the validation cohort, patients with scores 2 or 3 were more likely to achieve HBsAg seroclearance (Fig. 4c). Significantly few patients with a score of 0–1 achieved HBsAg seroclearance in the derivation and validation cohorts despite long-term NUC treatment for > 10 years.

### Longitudinal changes in the quantification of HBsAg distribution during long-term NUC treatment

We evaluated the distribution of on-treatment HBsAg levels per 5 years based on the baseline HBeAg status. The patients were categorized into three groups of HBsAg levels as follows: < 10, 10–100, and ≥ 100 IU/mL. The proportion of patients with HBsAg ≥ 100 IU/mL gradually decreased over time (Supplementary Fig. 7). In total, 70% of the patients with baseline HBeAg positivity still had HBsAg ≥ 100 IU/mL at year 15 (Supplementary Fig. 7a), and 48.7% of the patients with baseline HBeAg negativity had HBsAg ≥ 100 IU/mL at year 15 (Supplementary Fig. 7b). Similar findings were observed in the validation cohort (Supplementary Fig. 8). Despite > 15 years of NUC treatment, many patients did not achieve low levels of HBsAg in the pathway to HBsAg seroclearance in the derivation and validation cohorts.

## Discussion

Our study showed that on-treatment viral factors affected subsequent HBsAg seroclearance and reduction in patients treated with NUC for > 10 years. In contrast, patients without these favorable viral factors had almost no chance of achieving HBsAg seroclearance or reduction, even after prolonged NUC treatment. These findings suggest that new HBV therapies for patients without favorable on-treatment viral factors are required to achieve HBsAg seroclearance.

Despite long-term NUC treatment, few patients achieve HBsAg seroclearance [11, 12, 14, 18, 19]. However, a report covering an NUC treatment duration of > 10 years is remarkably limited. Furthermore, although previous reports have identified factors associated with HBsAg seroclearance, no reports have evaluated HBsAg seroclearance based on longitudinal changes in HBV markers for 5 years from the start of NUC as our current study. Using landmark analysis and time-dependent covariates, this study demonstrated that patients with baseline HBeAg positivity who achieved early HBeAg loss had higher rates of subsequent HBsAg seroclearance. In contrast, in patients with delayed HBeAg loss or persistent positivity for HBeAg, the rate of HBsAg seroclearance was extremely low, even after long-term NUC treatment of > 10 years. Even in patients who achieved HBeAg loss within the first 2 years, the annual HBsAg seroclearance rate was only 1.6%, which was not high. In the baseline HBeAg-negative cohort, the rate of HBsAg seroclearance was higher in patients who achieved HBsAg < 100 IU/mL within 5 years of the start of NUC treatment. In contrast, HBsAg seroclearance was limited to patients who did not achieve HBsAg < 100 IU/mL. Moreover, a substantial number of patients still had HBsAg ≥ 100 IU/mL even 15 years after the start of NUC. Moreover, we analyzed HBsAg < 10 IU/mL as another endpoint in the functional cure pathway, and the factors associated with this endpoint were similar to those associated with HBsAg seroclearance. In addition to viral factors, female sex had a negative effect on HBsAg seroclearance in baseline HBeAg-positive patients. We evaluated two large independent cohorts, and the results were similar in both. Therefore, many patients will not achieve a functional cure even with lifelong administration of NUC, early development of new treatments that can achieve a functional cure is desirable.

In this study, we analyzed the effects of different types of NUC compounds on HBsAg seroclearance and identified no differences in the seroclearance rate based on the type of first-line NUC. While early HBeAg loss was associated with subsequent HBsAg seroclearance in patients with HBeAg positivity, the factors associated with early HBeAg loss considerably differed from those associated with HBsAg seroclearance (Table 2, Supplementary Table 8). These findings suggest that HBeAg loss and subsequent HBsAg seroclearance are mediated by different mechanisms. In the validation cohort, elevated baseline ALT and the type of first-line NUC were also associated with early HBeAg loss. (Supplementary Table 8). Specifically, patients initially treated with ETV, TDF, or TAF as a first-line NUC were less likely to achieve early HBeAg loss in this cohort. This observation may be partly explained by the increased incidence of breakthrough hepatitis associated with lamivudine resistance and its rescue therapy by adefovir, a drug associated with HBeAg loss and seroconversion [20]. Importantly, these factors were associated with early HBeAg but not HBsAg seroclearance. In addition, we observed no significant difference in early HBeAg loss between patients treated with ETV and those treated with TDF/TAF (*P* = 0.555, data not shown). However, these findings should be interpreted cautiously, as treatment switching may complicate the analysis.

The mechanisms underlying NUC-induced HBeAg and HBsAg seroclearance remain unclear. The NUC compounds themselves have no immunological effects. However, the host immune response to HBV before treatment might be related to early HBeAg seroconversion [21–23]. Moreover, a decrease in intrahepatic HBV levels leads to recovery of the T-cell response [24, 25]. Changes in cytokines during NUC treatment are associated with HBsAg seroclearance, suggesting that changes in the host immune response during NUC treatment may be associated with a functional cure [25–27]. In this study, the higher rates of subsequent HBsAg seroclearance in patients who achieved early HBeAg loss may have been due to rapid recovery of the host immune response to HBV. However, immunological assessments were not performed in this study. Therefore, conducting immunological studies on long-term NUC-treated patients is necessary.

Several studies have been conducted on the discontinuation of NUC treatment to reduce its long-term burden [28]. Among these studies, clinical relapse after discontinuation is low and HBsAg seroclearance after discontinuation is higher in patients with HBsAg < 100 IU/mL [29–31]. Furthermore, combining low HBsAg with low HBcrAg levels or undetectable HBV RNA enables a more accurate prediction of successful NUC discontinuation [29, 32, 33]. Considering this, even with NUC treatment for > 10 years, the number of patients who can achieve HBsAg < 100 IU/mL seems to be relatively limited, as shown in the current data. Close monitoring is necessary after the time being after discontinuation of NUCs to prevent hepatic decompensation due to severe clinical relapse. Therefore, many patients may need to continue NUC treatment while awaiting new HBV treatments to become available.

We developed a simple prediction model (SLOPES50) for HBsAg seroclearance using baseline age, sex, and earlier HBeAg loss in baseline HBeAg-positive patients. This model had good predictive capability for HBsAg seroclearance in the validation cohort and may be expected to be used in clinical settings. Cumulative HBsAg seroclearance rates were extremely low in patients with a score of 0–1. This finding indicates that female patients aged < 50 years have almost no chance of a functional cure if they achieve HBeAg loss within 3 years. Female sex was associated with low rates of spontaneous HBsAg seroclearance in a large cohort [34]. However, the relationship between sex and NUC-induced HBsAg seroclearance remains unclear, as female sex was not associated with early HBeAg loss in the present study. Therefore, further research is required to establish this relationship. Moreover, the SLOPES50 model requires prior HBeAg loss, we could not calculate this score at baseline. We believe that it is clinically useful to calculate this score for patients treated with NUC for a certain period. For those patients, on-treatment SLOPES50 scores and HBsAg level at that time, may help identify individuals with a higher likelihood of achieving HBsAg seroclearance and thus may be useful selecting candidates for future HBV therapies aiming at a functional cure.

New treatments for HBV infection based on various mechanisms of action are currently under development [15]. Some of them have progressed to phase 2 or 3 clinical trials [15]. However, even with bepirovirsen, only approximately 10% of participants achieved HBsAg seroclearance when used in combination with NUC treatment. [16] Furthermore, the effect can only be expected in patients with low HBsAg levels, and a functional cure was rarely achieved in HBeAg-positive patients.

This study has certain limitations. First, it was a retrospective study. Therefore, there may have been a potential selection bias. However, we ensured the replicability of the analysis results using another independent cohort. Second, a lack of HBsAg data exists in some cases. Third, the HBV genotype could not be assessed. As previous studies have shown an association between HBV genotype and HBsAg, we need to investigate them in the future. Fourth, in this study, a slight difference in the crude rates of HBsAg seroclearance was observed between the derivation and validation cohorts, particularly among patients with baseline HBeAg negativity. Among baseline factors, the derivation cohort included slightly older patients, with relatively lower ALT levels and fewer cases with a prior history of IFN than the validation cohort (Table 1 and Supplementary Table 1). This discrepancy likely reflects differences in physician decision-making regarding NUC or IFN initiation at each facility. Nevertheless, the results derived from the derivation and validation cohorts were highlight consistent, indicating that the findings of this study are generalizable. Finally, new markers, including HBcrAg and HBV RNA, which have recently attracted attention for their clinical utility as novel HBV biomarkers [35, 36], were not evaluated.

Our study highlighted that on-treatment viral factors affected subsequent HBsAg seroclearance and reduction in patients treated with long-term NUC for > 10 years in two independent cohorts. A simple prediction model (SLOPES) for NUC-induced HBsAg seroclearance was created using sex and earlier HBeAg loss. If favorable HBV marker changes as described above are not achieved within 5 years from baseline, the probability of achieving a functional cure thereafter seems to be relatively low. The decrease in HBsAg levels during NUC treatment is passive; however, measuring HBsAg levels can predict the achievement of a functional cure thereafter. New treatments for HBV infection will be developed in the future for patients for whom a functional cure cannot be expected with NUC treatment alone.

## Supplementary Information

Below is the link to the electronic supplementary material.

Supplementary file1 (DOCX 1351 KB)

## Abbreviations

HBeAg: Hepatitis B e-antigen
HBV: Hepatitis B virus
HBsAg: Hepatitis B surface antigen
GGTP: Gamma-glutamyltransferase
ALT: Alanine aminotransferase
NUC: Nucleos(t)ide analog
ETV: Entecavir
LAM: Lamivudine
TAF: Tenofovir alafenamide
TDF: Tenofovir disoproxil fumarate
IFN: Interferon
HR: Hazard ratio
