# Supplementary material for: On-treatment viral factors affect subsequent hepatitis B surface antigen seroclearance in patients treated with nucleos(t)ide analogs for > 10 years
Source: J Gastroenterol. 2026 Apr 21;61(7):990–1002. doi: 10.1007/s00535-026-02415-3 (PMC13283148; doi:10.1007/s00535-026-02415-3)
Supplement: Supplementary file 1 — Supplementary file1 (DOCX 1351 KB) [file 535_2026_2415_MOESM1_ESM.docx]

**Supplementary Information**

**On-treatment viral factors affect subsequent hepatitis B surface antigen seroclearance in patients treated with nucleos(t)ide analogs for >10 years**

Tetsuya Hosaka, MD,^1,2^ Hayato Hikita, MD. PhD,^1^ Yuki Tahata, MD. PhD,^1^ Ryoko Yamada, MD. PhD,^1^ Kazuhiro Murai, MD. PhD,^1^ Masanori Miyazaki, MD. PhD,^3^ Hisashi Ishida, MD. PhD,^4^ Atsushi Hosui, MD. PhD,^5^ Ryotaro Sakamori, MD. PhD,^6^ Nobuyuki Tatsumi, MD. PhD,^7^ Yoshinori Doi, MD. PhD,^8^ Kazuyoshi Ohkawa, MD. PhD,^9^ Satoshi Egawa, MD. PhD,^10^ Takatoshi Nawa, MD. PhD,^11^ Yasutoshi Nozaki, MD. PhD,^12^ Kazuho Imanaka, MD. PhD,^13^ Masanori Nakahara, MD. PhD,^14^ Mitsuru Sakakibara, MD. PhD,^15^ Takayuki Yakushijin, MD. PhD,^16^ Yuichi Yoshida, MD. PhD,^17^ Hiroyuki Ogawa, MD. PhD,^18^ Takeo Usui, MD. PhD,^19^ Kengo Matsumoto, MD. PhD,^20^ Kazuki Maesaka, MD. PhD,^1^ Kumiko Shirai, MD. PhD,^1^ Yuki Makino, MD. PhD,^1^ Yoshinobu Saito, MD. PhD,^1^ Takahiro Kodama, MD. PhD,^1^ Tetsuo Takehara MD. PhD,^12^

^1^Department of Gastroenterology and Hepatology, The University of Osaka Graduate School of Medicine, Suita, Japan

^2^Department of Hepatology, Toranomon Hospital, Tokyo, Japan

^3^Department of Gastroenterology and Hepatology, Osaka Police Hospital

^4^Department of Gastroenterology and Hepatology, Ikeda Municipal Hospital

^5^Department of Gastroenterology and Hepatology, Osaka Rosai Hospital

^6^Department of Gastroenterology and Hepatology, NHO Osaka National Hospital

^7^Department of Gastroenterology and Hepatology, Japan Community Healthcare Organization Osaka Hospital

^8^Department of Gastroenterology and Hepatology, Otemae Hospital

^9^Department of Hepatobiliary and Pancreatic Oncology, Osaka International Cancer Institute, Osaka

^10^Department of Gastroenterology and Hepatology, Kinki Central Hospital of Mutual Aid Association of Public School Teachers

^11^Department of Gastroenterology and Hepatology, Higashiosaka City Medical Center

^12^Department of Gastroenterology and Hepatology, Kansai Rosai Hospital

^13^Department of Gastroenterology and Hepatology, Itami City Hospital

^14^ Department of Gastroenterology and Hepatology, Minoh City Hospital

^15^Department of Gastroenterology and Hepatology, Yao Municipal Hospital

^16^Department of Gastroenterology and Hepatology, Osaka General Medical Center

^17^Department of Gastroenterology and Hepatology, Suita Municipal Hospital

^18^Department of Gastroenterology, Nishinomiya Municipal Central Hospital

^19^Department of Gastroenterology and Hepatology, Ashiya Municipal Hospital

^20^Department of Gastroenterology and Hepatology, Toyonaka Municipal Hospital

**MATERIALS AND METHODS**

**Study population and clinical data collection**

Patients enrolled in this study were chronically mono-infected with HBV and had a confirmed HBsAg-positive status for at least 6 months, with no history of HCC. NUC treatment was initiated when a patient presented with abnormal ALT levels (>30 IU/I) and elevated HBV DNA levels (≥ 4 log copies/mL). Patients with HBV infection and advanced fibrosis were treated with NUCs if their ALT levels were normal, whereas those without fibrosis or with normal HBV DNA/ALT levels were not. Of the 946 initially recruited patients, 103 were excluded for the following reasons: hepatitis C co-infection, use of NUCs for the prophylaxis of HBV reactivation under immunosuppressive or cancer chemotherapy, and insufficient clinical and HBV markers. The remaining 843 patients were included in the final analysis (Supplementary Figure 2).

All patients were regularly followed up at 1- to 3-month intervals, during which biochemical and serum HBV viral markers, including HBV DNA, HBeAg and quantification of HBsAg (qHBsAg), blood counts, tumor markers (alpha-fetoprotein and des-γ-carboxyl prothrombin), and cirrhosis and HCC statuses, were monitored as a routine monitoring. Cirrhosis was diagnosed via laparoscopy, liver biopsy, imaging modalities, or portal hypertension, based on the criteria of each institution. All patients underwent ultrasonography, helical dynamic computed tomography, or magnetic resonance imaging at intervals of 3–6 months for those with cirrhosis and 6–12 months for those without cirrhosis.

**Hepatitis B virus markers**

HBsAg levels were measured, including the ARCHITECT HBsAg QT assay kits (Abbott Laboratories, Tokyo, Japan), which have lower and upper limits of detection of 0.05 and 250 IU/mL, respectively, or the ECLusys HBsAg II quant II assay kit (Roche Diagnostics, Tokyo, Japan) with 0.05–13,000 IU/mL of its quantification range. In some cases, to expand the upper range from 250 to 125,000 IU/mL, serum samples with off-scale values were diluted with ARCHITECT diluents, according to the manufacturer’s instructions. HBV DNA was quantified using the COBAS® Amplicor HBV Monitor Test (Roche Diagnostics, Tokyo, Japan), which has a dynamic range of >2.6–7.6 log copies/mL, or the COBAS® TaqMan HBV Test version 2.0 (Roche Diagnostics, Tokyo, Japan), which has a dynamic range of >2.1–9.0 log copies/mL. HBeAg positivity was determined using commercially available assay kits (Architect HBeAg, Abbott Laboratories; Lumipulse G, Fujirebio Inc., Tokyo, Japan; ECLusys HBeAg, Roche Diagnostics, Tokyo, Japan).

**Results**

**Patient characteristics**

The baseline characteristics of the patients in the validation cohort are shown in Supplementary Table 1. The HBeAg+ and HBeAg– cohorts comprised 905 and 876 patients, respectively. During respective median follow-up durations of 12.0 and 11.3 years, 56 (6.2%) patients in the HBeAg+ cohort (5.3/1,000 person-years) and 93 (10.6%) patients in the HBeAg– cohort achieved HBsAg seroclearance (9.5/1,000 person-years).

**Association between on-treatment HBeAg loss and HBsAg seroclearance or achieving HBsAg <10 IU/mL in patients with baseline HBeAg positivity**

The 10- and 15-year HBsAg seroclearance rates were 6.5% and 10.4% in patients who achieved HBeAg loss before year 3 and 1.6% and 1.6% in patients whose HBeAg remained positive at year 3, respectively. In the landmark-analysis at year 5, the 10- and 15-year HBsAg seroclearance rates were 2.2% and 5.4% in patients who achieved HBeAg loss before year 5 and 0.9% and 0.9% in patients whose HBeAg remained positive at year 5, respectively. Regarding the endpoint of achieving HBsAg <10 IU/mL, the 10- and 15-year incidence rates were 8.8% and 28.6% in patients who achieved HBeAg loss before year 3 and 3.6% and 11.1% in patients whose HBeAg remained positive at year 3, respectively. Multivariate analysis in the validation cohort revealed that HBeAg loss before year 3 or 5 was significantly associated with HBsAg seroclearance and a reduction of <10 IU/mL (Supplementary Table 6a and b).

**Association between on-treatment HBsAg levels and HBsAg seroclearance or achieving HBsAg <10 IU/mL in patients with baseline HBeAg negativity**

The 10- and 15-year HBsAg seroclearance rates were 30.3% and 30.3% in patients with HBsAg <100 IU/mL at year 3 and 1.7% and 1.7% in patients without it, respectively. The 10- and 15-year HBsAg seroclearance rates were 19.4% and 19.4% in patients with HBsAg <100 IU/mL at year 5 and 0.6% and 0.6% in patients without it, respectively. In the landmark analysis of the validation cohort, HBsAg seroclearance or reduction was also likely to occur in patients with HBsAg <100 IU/mL at year 3 or 5 (Supplementary Figure 5a–d).

Multivariate analysis in the validation cohort revealed that HBsAg <100 IU/mL at year 3 or 5 were significantly associated with HBsAg seroclearance and a reduction of <10 IU/mL after the same adjustment (Supplementary Table 7a and b).

**Likelihood of HBsAg seroclearance or HBsAg reduction after earlier HBeAg loss**

In the derivation cohort, the annual rates (95% CI) of HBsAg seroclearance were 0.016 (0.010–0.027) per person-year in patients with HBeAg loss from baseline to year 2, 0.004 (0.001–0.013) for patients with HBeAg loss from year 2 to 4, 0.004 (0.001–0.010) for patients with HBeAg loss after year 4, and 0.000 for patients without HBeAg loss (Figure 3a). The annual rates (95% CI) of achieving HBsAg <10 IU/mL were 0.026 (0.017–0.039) per person-year in patients with HBeAg loss from baseline to year 2, 0.015 (0.008–0.027) per person-year in patients with HBeAg loss from year 2 to 4, 0.010 (0.005–0.019) per person-year in patients with HBeAg loss after year 4, and 0.001 (0.000–0.006) per person-year in patients without HBeAg loss (Figure 3b).

| List of Osaka Liver Forum (OLF) | Number of enrolled patients |
| --- | --- |
| The University of Osaka, Graduate School of Medicine | 153 |
| Osaka Police Hospital | 146 |
| Ikeda Municipal Hospital | 82 |
| Osaka Rosai Hospital | 77 |
| NHO Osaka National Hospital | 71 |
| Japan Community Healthcare Organization Osaka Hospital | 51 |
| Otemae Hospital | 44 |
| Osaka International Cancer Institute | 38 |
| Kinki Central Hospital of Mutual Aid Association of Public School Teachers | 32 |
| Higashiosaka City Medical Center | 31 |
| Kansai Rosai Hospital | 22 |
| Itami City Hospital | 21 |
| Minoh City Hospital | 18 |
| Yao Municipal Hospital | 15 |
| Osaka General Medical Center | 14 |
| Suita Municipal Hospital | 12 |
| Nishinomiya Municipal Central Hospital | 10 |
| Ashiya Municipal Hospital | 4 |
| Toyonaka Municipal Hospital | 2 |
| Total | 843 |

**Supplementary Figure 1.** List of Osaka Liver Forum (OLF) (Derivation cohort)


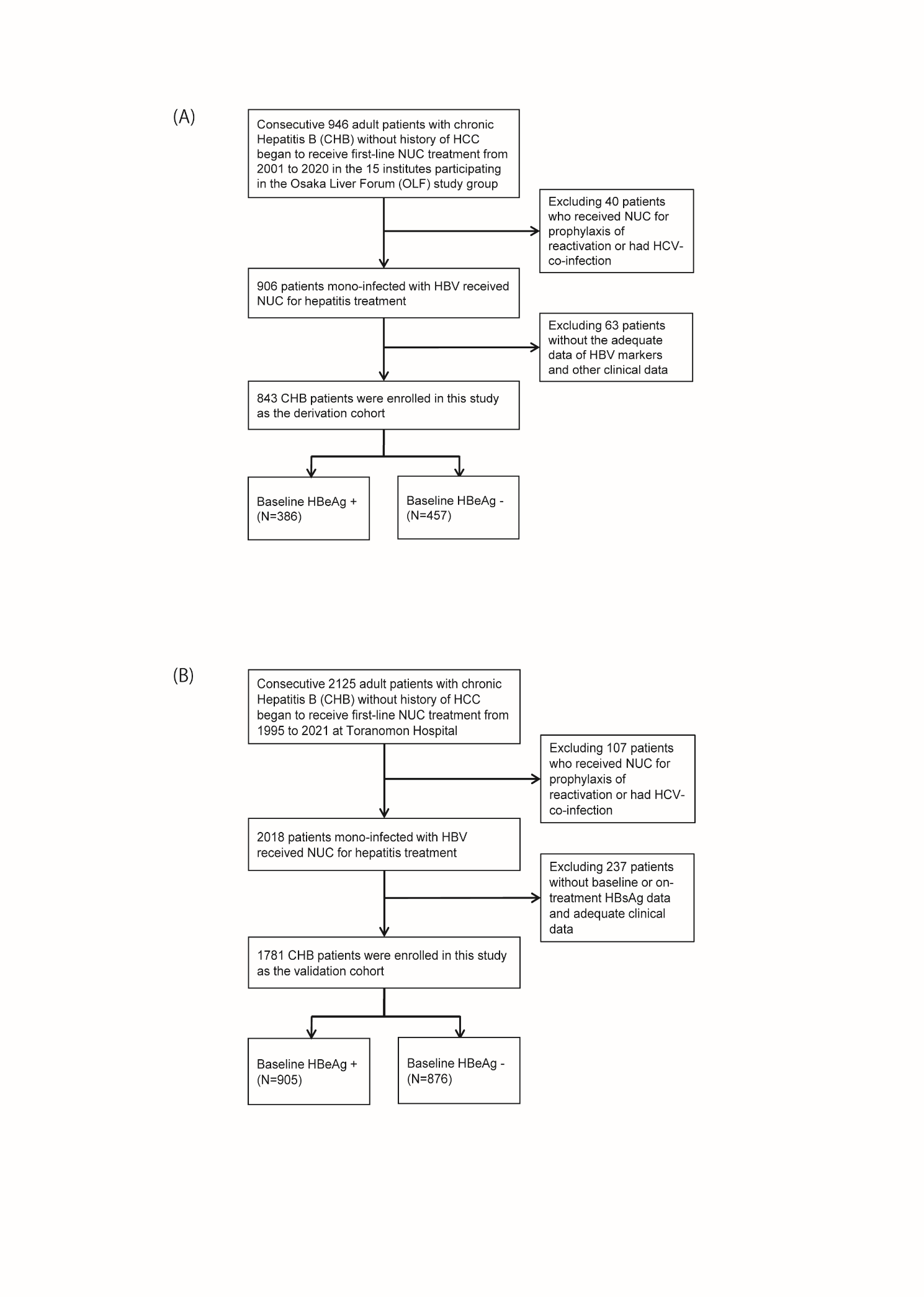


**Supplementary Figure 2.** Selection of the study participants. (A) Derivation cohort (B) Validation cohort

| Characteristic | HBeAg positive  (N=905) | HBeAg negative  (N=876) | All (N=1781) | Missing, no (%) |
| --- | --- | --- | --- | --- |
| Age (y) | 39 (33-48) | 50 (41-58) | 44 (36-54) | 0 |
| Gender (male), no (%) | 642 (70.9%) | 591 (67.5%) | 1233 (69.2%) | 0 |
| Cirrhosis, no (%) | 153 (16.9%) | 179 (20.4%) | 332 (18.6%) | 0 |
| HBeAg positive, no (%) | - | - | 905 (50.8%) | 0 |
| HBV DNA (log IU/mL) | 7.2 (6.1-7.9) | 5.0 (3.6-6.0) | 6.1 (4.7-7.2) | 0 |
| HBsAg > 250 IU/mL, no (%) | 853 (94.3%) | 718 (82.0%) | 1571 (88.2%) | 0 |
| ALT level (IU/L) | 110 (61-272) | 70 (35-153) | 59 (46–200) | 0 |
| GGTP level (IU/L) | 53 (31-99) | 39 (24-73) | 45 (27–86) | 2 (0.1%) |
| Serum albumin (g/L) | 3.8 (3.6-4.1) | 4.0 (3.7-4.2) | 3.9 (3.6–4.1) | 0 |
| Platelet (10^5^/mm^3^) | 17.2 (13.5-21.1) | 16.9 (13.7-20.5) | 17.0 (13.7-20.8) | 0 |
| Prior history of IFN Tx, no (%) | 280 (30.9%) | 188 (21.5%) | 468 (26.3%) | 0 |
| First-line NUC  (ETV: LAM: TAF: TDF) | 366: 408: 67: 64 | 449: 305: 73: 49 | 815: 713: 140: 110 | 0 |
| Treatment duration (y) | 12.0 (5.9-17.0) | 11.3 (6.2-15.6) | 11.6 (6.1-16.4) |  |
| HBsAg seroclearance cases, no | 56 (5.3/1000 PY) | 93 (9.5/1000 PY) | 149 (7.3/1000 PY) |  |

**Supplementary Table 1.** Baseline characteristics in the validation cohort.

All values are expressed as medians (25th to 75th percentile) or numbers (percentage of total, %).

Abbreviations: HBeAg, hepatitis B e antigen; HBsAg, hepatitis B surface antigen; ALT, ; GGTP, gamma glutamyltransferase; IFN, interferon; NUC, nucleos(t)ide analogue; ETV, entecavir; LAM, lamivudine; TAF, tenofovir alafenamide; TDF, tenofovir disoproxil fumerate; PY, patient years


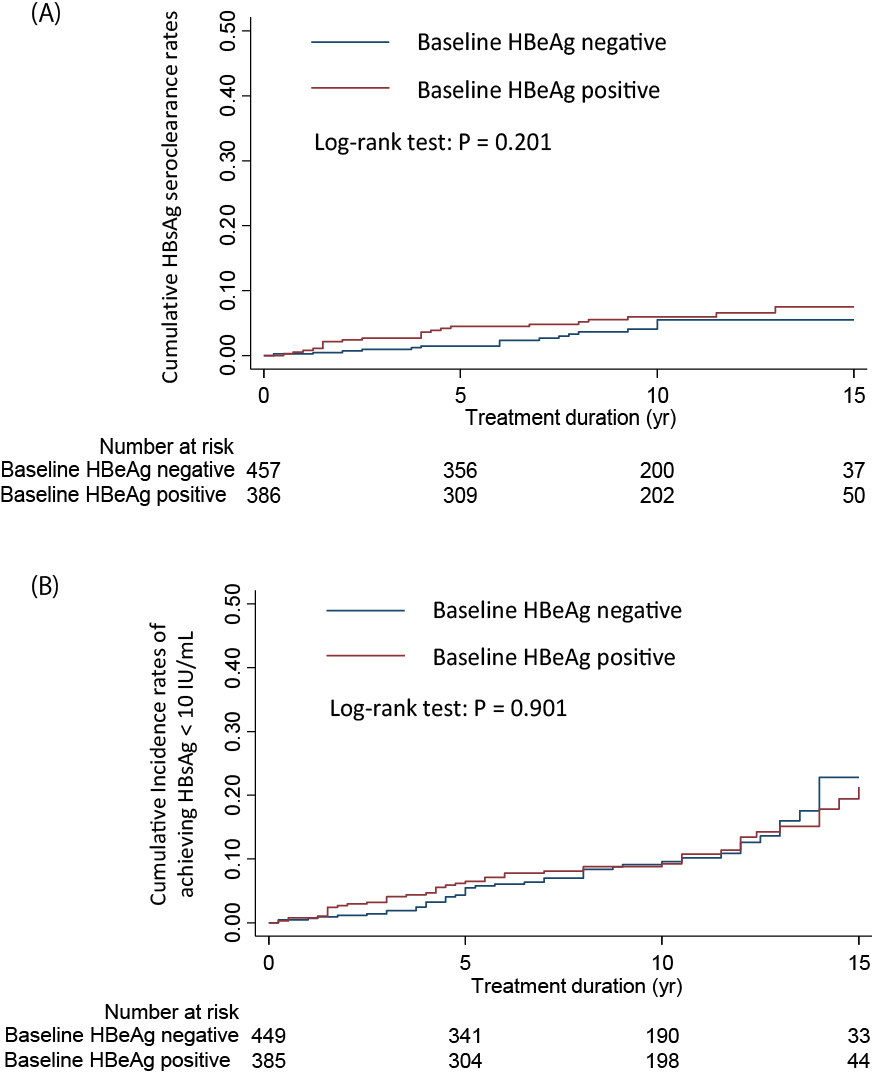


**Supplementary Figure 3.** Kaplan-Meier curves that represented the cumulative rates of HBsAg seroclearance or achieving HBsAg < 10 IU/mL according to baseline HBeAg status in the derivation cohort. (A) HBsAg seroclearance (B) achieving HBsAg < 10 IU/mL.

(A)

| Variable | Univariable HR (95% CI) | P | †Multivariable adjusted HR (95% CI) | P | *Multivariable adjusted HR (95% CI) | P |
| --- | --- | --- | --- | --- | --- | --- |
| **Age (y)** | 1.02 (0.99-1.04) | 0.221 |  |  |  |  |
| **Gender (male)** | 2.14 (1.01-4.54) | 0.046 | 2.60 (1.00-6.75) | 0.049 | 2.64 (1.02-6.80) | 0.045 |
| **Cirrhosis** | 0.69 (0.31-1.52) | 0.355 |  |  |  |  |
| **HBV DNA (log IU/mL)** | 1.01 (0.84-1.22) | 0.922 |  |  |  |  |
| **HBsAg > 250 IU/mL** | 0.68 (0.29-2.24) | 0.809 |  |  |  |  |
| **ALT level (IU/L)** | 1.001  (1.001-1.002) | < 0.001 |  |  |  |  |
| **GGTP level (IU/L)** | 1.005  (1.003-1.007) | < 0.001 |  |  |  |  |
| **Serum albumin (g/L)** | 0.45 (0.24-0.85) | 0.014 |  |  |  |  |
| **Platelet (10^5/mm3)** | 0.98 (0.93-1.02) | 0.321 |  |  |  |  |
| **Prior history of IFN Tx** | 1.97 (1.17-3.34) | 0.011 |  |  |  |  |
| **First-line NUC**  **LAM**  **ETV**  **TDF or TAF** | 1  0.61 (0.32-1.17)  1.45 (0.53-3.94) | Ref.  0.138  0.471 |  |  |  |  |
| **On-treatment HBeAg loss (Time-dependent covariate)** | 9.88 (3.37-28.9) | < 0.001 | 8.41 (2.86-24.8) | < 0.001 | 9.53 (3.25-28.0) | < 0.001 |

(B)

| Variable | Univariable HR (95% CI) | P | †Multivariable Adjusted HR (95% CI) | P | *Multivariable Adjusted HR (95% CI) | P |
| --- | --- | --- | --- | --- | --- | --- |
| **Age (y)** | 1.03 (1.01-1.04) | 0.010 |  |  | 1.02 (1.00-1.04) | 0.042 |
| **Gender (male)** | 3.01 (1.51-6.02) | 0.002 | 4.21 (1.79-9.89) | 0.001 |  |  |
| **Cirrhosis** | 1.36 (0.81-2.30) | 0.251 |  |  |  |  |
| **HBV DNA (log IU/mL)** | 0.90 (0.78-1.03) | 0.129 |  |  |  |  |
| **HBsAg > 250 IU/mL** | 0.33 (0.18-0.62) | < 0.001 | 0.36 (0.19-0.67) | 0.001 | 0.31 (0.17-0.58) | < 0.001 |
| **ALT level (IU/L)** | 1.001 (1.000-1.001) | 0.015 |  |  |  |  |
| **GGTP level (IU/L)** | 1.004 (1.002-1.006) | < 0.001 |  |  |  |  |
| **Serum albumin (g/L)** | 0.42 (0.25-0.72) | 0.002 |  |  |  |  |
| **Platelet (10^5/mm3)** | 0.94 (0.91-0.98) | 0.003 |  |  |  |  |
| **Prior history of IFN Tx** | 1.45 (0.94-2.24) | 0.095 |  |  |  |  |
| **First-line NUC**  **LAM**  **ETV**  **TDF or TAF** | 1  0.70 (0.43-1.14)  0.98 (0.38-2.55) | Ref.  0.151  0.966 |  |  |  |  |
| **On-treatment HBeAg loss (Time-dependent covariate)** | 5.51 (2.68-11.3) | < 0.001 | 5.07 (2.44-10.5) | < 0.001 | 5.62 (2.74-11.5) | < 0.001 |

**Supplementary Table 2.** (A) Factors associated with HBsAg seroclearance among patients with baseline HBeAg positivity in the validation cohort using time-dependent model（Cox proportional hazard regression）

†Adjusted for gender, ALT, GGTP, serum albumin, prior history of IFN and on-treatment HBeAg loss

*Adjusted for the factors associated with HBsAg seroclearance in the derivation cohort including age, gender, platelet and on-treatment HBeAg loss

(B) Factors associated with achieving HBsAg <10 IU/mL among patients with baseline HBeAg positivity in the validation cohort using time-dependent model（Cox proportional hazard regression））

†Adjusted for age, gender, ALT, GGTP, serum albumin, platelet and on-treatment HBeAg loss

*Adjusted for the factors associated with HBsAg seroclearance in the derivation cohort including age, gender, platelet and on-treatment HBeAg loss

**(A)**

| Variable | Univariate HR (95% CI) | P | Multivariate-adjusted HR (95% CI)^†^ | P |
| --- | --- | --- | --- | --- |
| **Age (y)** | 1.04 (1.02-1.07) | 0.001 | 1.05 (1.01-1.08) | 0.005 |
| **Sex (male), no. (%)** | 1.85 (0.94-3.64) | 0.077 |  |  |
| **Cirrhosis, no. (%)** | 1.27 (0.64-2.50) | 0.494 |  |  |
| **HBV DNA (log copies/mL)** | 0.85 (0.66-1.08) | 0.184 |  |  |
| **HBsAg >250 IU/mL** | 0.15 (0.07-0.35) | <0.001 | 0.11 (0.04-0.29) | <0.001 |
| **ALT level (IU/L)** | 1.01 (0.97-1.05) | 0.801 |  |  |
| **GGTP level (IU/L)** | 0.998 (0.992-1.003) | 0.382 |  |  |
| **Serum albumin (g/L)** | 0.49 (0.29-0.83) | 0.008 |  |  |
| **Platelet (10^5^/mm^3^)** | 1.04 (0.99-1.09) | 0.108 |  |  |
| **History of IFN Tx** | 1.42 (0.70-2.88) | 0.332 |  |  |
| **First-line NUC**  **LAM**  **ETV**  **TDF or TAF** | 1  0.98 (0.49-1.95)  4.13 (0.85-20.11) | Ref.  0.955  0.079 |  |  |
| **On-treatment HBeAg loss (time-dependent covariate)** | 13.1 (4.85-35.4) | < 0.001 | 14.9 (4.77-46.4) | < 0.001 |

^†^Adjusted for age, sex, albumin, and on-treatment HBeAg loss

**(B)**

| Variable | Univariate HR (95% CI) | P | Multivariate-adjusted HR (95% CI)^‡^ | P |
| --- | --- | --- | --- | --- |
| **Age (y)** | 1.03 (0.99–1.06) | 0.100 |  |  |
| **Sex (male), no. (%)** | 1.19 (0.68–2.07) | 0.543 |  |  |
| **Cirrhosis, no. (%)** | 1.17 (0.62–2.20) | 0.629 |  |  |
| **HBV DNA (log copies/mL)** | 0.79 (0.66–0.95) | 0.011 |  |  |
| **HBsAg >250 IU/mL** | 0.06 (0.03–0.12) | <0.001 | 0.06 (0.03–0.13) | <0.001 |
| **ALT level (IU/L)** | 1.00 (0.96–1.04) | 0.962 |  |  |
| **GGTP level (IU/L)** | 1.002 (1.000–1.004) | 0.060 |  |  |
| **Serum albumin (g/L)** | 1.08 (0.61–1.93) | 0.792 |  |  |
| **Platelet (105/mm^3^)** | 0.98 (0.93–1.03) | 0.388 |  |  |
| **History of IFN Tx** | 1.15 (0.52–2.56) | 0.728 |  |  |
| **First-line NUC**  **LAM**  **ETV**  **TDF or TAF** | 1  1.73 (0.77-3.90)  3.99 (0.94-17.03) | Ref.  0.188  0.062 |  |  |

^‡^Adjusted for HBV DNA and HBsAg

**Supplementary Table 3.** Factors associated with achieving HBsAg <10 IU/mL according to baseline HBeAg status in the time-dependent model in the derivation cohort. (A) Baseline HBeAg-positive (B) Baseline HBeAg-negative

(A)

| Variable | Univariable HR (95% CI) | P | *Multivariable adjusted HR (95% CI) | P |
| --- | --- | --- | --- | --- |
| **Age (y)** | 1.00 (0.98-1.02) | 0.953 |  |  |
| **Gender (male), no (%)** | 1.42 (0.85-2.36) | 0.176 |  |  |
| **Cirrhosis, no (%)** | 0.80 (0.48-1.33) | 0.386 |  |  |
| **HBV DNA (log copies/mL)** | 0.78 (0.69-0.88) | < 0.001 | 0.87 (0.76-0.99) | 0.035 |
| **HBsAg > 250 IU/mL** | 0.25 (0.16-0.37) | < 0.001 | 0.29 (0.19-0.46) | < 0.001 |
| **ALT level (IU/L)** | 1.000 (1.000-1.001) | 0.327 |  |  |
| **GGTP level (IU/L)** | 0.999 (0.996-1.002) | 0.463 |  |  |
| **Serum albumin (g/L)** | 1.81 (0.96-3.39) | 0.065 |  |  |
| **Platelet (10<sup>5</mm3)** | 1.02 (0.98-1.06) | 0.386 |  |  |
| **Prior history of IFN Tx** | 1.11 (0.71-1.74) | 0.646 |  |  |
| **First-line NUC**  **LAM**  **ETV**  **TDF or TAF** | 1  0.88 (0.56-1.40)  0.71 (0.16-3.04) | Ref.  0.593  0.641 |  |  |

(B)

| Variable | Univariable HR (95% CI) | P | †Multivariable adjusted HR (95% CI) | P |
| --- | --- | --- | --- | --- |
| **Age (y)** | 1.00 (0.98-1.02) | 0.897 |  |  |
| **Gender (male), no (%)** | 1.40 (0.91-2.16) | 0.130 |  |  |
| **Cirrhosis, no (%)** | 1.09 (0.72-1.64) | 0.692 |  |  |
| **HBV DNA (log copies/mL)** | 0.83 (0.74-0.92) | 0.001 |  |  |
| **HBsAg > 250 IU/mL** | 0.25 (0.17-0.37) | < 0.001 | 0.29 (0.19-0.43) | < 0.001 |
| **ALT level (IU/L)** | 1.000 (1.000-1.001) | 0.122 |  |  |
| **GGTP level (IU/L)** | 1.002 (1.000-1.004) | 0.035 | 1.003 (1.000-1.005) | 0.017 |
| **Serum albumin (g/L)** | 1.83 (1.07-3.14) | 0.028 | 1.69 (1.00-2.83) | 0.049 |
| **Platelet (10<sup>5/mm3)** | 1.00 (0.97-1.04) | 0.983 |  |  |
| **Prior history of IFN Tx** | 1.13 (0.77-1.68) | 0.528 |  |  |
| **First-line NUC**  **LAM**  **ETV**  **TDF or TAF** | 1  0.84 (0.58-1.21)  0.81 (0.32-2.07) | Ref.  0.351  0.656 |  |  |

**Supplementary Table 4.** Factors associated with HBsAg seroclearance and achieving HBsAg < 10 IU/mL among patients with baseline HBeAg negativity in the validation cohort. (A) HBsAg seroclearance (B) Achieving HBsAg < 10 IU/mL

*Adjusted for HBV DNA and HBsAg

†Adjusted for HBV DNA, HBsAg, GGTP and serum albumin


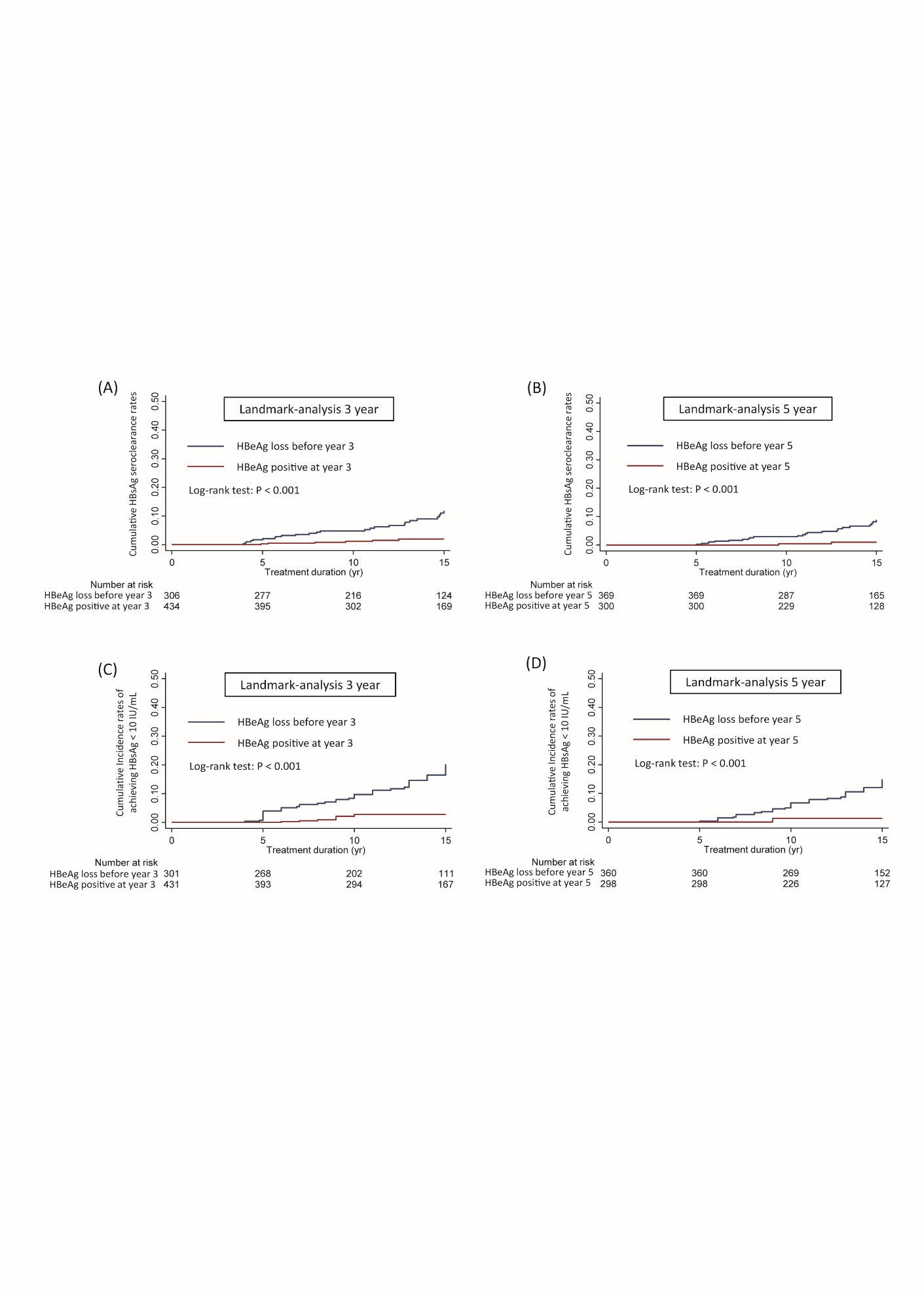


**Supplementary Figure 4.** Cumulative incidence rates of HBsAg seroclearance or achieving HBsAg < 10 IU/mL using the landmark-analysis among patients with baseline HBeAg positivity in the validation cohort (A) HBsAg seroclearance by HBeAg status at year 3. The log-rank test revealed a statistically significant difference in the HBsAg seroclearance between patients with HBeAg loss before year 3 and without HBeAg loss (log-rank test: P < 0.001). (B) HBsAg seroclearance by HBeAg status at year 5. The log-rank test revealed a statistically significant difference in the HBsAg seroclearance between patients with HBeAg loss before year 5 and without HBeAg loss (log-rank test: P < 0.001). (C) achieving HBsAg < 10 IU/mL by HBeAg status at year 3. The log-rank test revealed a statistically significant difference in the achievement of HBsAg < 10 IU/mL between patients with HBeAg loss before year 3 and without HBeAg loss (log-rank test: P < 0.001). (D) achieving HBsAg < 10 IU/mL by HBeAg status at year 5. The log-rank test revealed a statistically significant difference in the achievement of HBsAg < 10 IU/mL between patients with HBeAg loss before year 5 and without HBeAg loss (log-rank test: P < 0.001).

1. Baseline HBeAg-positive

| Variable | | Total number | Cases | PY | Annual incidences/1000 PY (95% CI) | Adjusted HR (95% CI) † | P |
| --- | --- | --- | --- | --- | --- | --- | --- |
| **HBeAg loss before year 3** | **No** | 220 | 11 | 2327.3 | 4.73 (2.62–8.53) | 1 | - |
| **HBeAg loss before year 3** | **Yes** | 112 | 19 | 1256.9 | 15.1 (9.64–23.7) | 2.76 (1.30–5.88) | 0.009 |
| **HBeAg loss before year 5** | **No** | 161 | 5 | 1788.4 | 2.80 (1.16–6.71) | 1 | - |
| **HBeAg loss before year 5** | **Yes** | 141 | 17 | 1668.8 | 10.2 (6.33–16.4) | 3.16 (1.15–8.65) | 0.025 |

†Adjusted for age and HBeAg loss

1. Baseline HBeAg-negative

| Variable | | Total number | Cases | PY | Annual incidences/1000 PY (95% CI) | Adjusted HR (95% CI) ^‡^ | P |
| --- | --- | --- | --- | --- | --- | --- | --- |
| **HBsAg <100 IU/mL at year 3** | **No** | 327 | 16 | 3393.6 | 4.71 (2.89–7.70) | 1 | - |
|  | **Yes** | 36 | 19 | 240.1 | 79.2 (5.05–124.1) | 129.8 (22.8–738.7) | <0.001 |
| **HBsAg <100 IU/mL at year 5** | **No** | 287 | 11 | 3187.7 | 3.45 (1.91–6.23) | 1 | - |
|  | **Yes** | 27 | 11 | 239.3 | 46.0 (25.5–83.0) | 10.9 (3.14–37.9) | <0.001 |

^‡^Adjusted for baseline and on-treatment HBsAg

**Supplementary Table 5.** Association between on-treatment viral factors and achieving HBsAg < 10 IU/mL among patients with baseline HBeAg positivity and negativity in the derivation cohort (Landmark analysis)

1. HBsAg seroclearance

| Variable | | Total number | Cases | PY | Annual incidences/1000 PY (95% CI) | Adjusted HR (95% CI) † | P |
| --- | --- | --- | --- | --- | --- | --- | --- |
| **HBeAg loss before year 3** | **No** | 434 | 7 | 5550.4 | 1.26 (0.60-2.64) | 1 | - |
| **HBeAg loss before year 3** | **Yes** | 306 | 29 | 4063.9 | 7.14 (4.95-10.3) | 5.57 (2.44-12.7) | < 0.001 |
| **HBeAg loss before year 5** | **No** | 300 | 3 | 4084.7 | 0.73 (0.24-2.27) | 1 | - |
| **HBeAg loss before year 5** | **Yes** | 369 | 27 | 5226.3 | 5.17 (3.54-7.53) | 6.93 (2.10-22.8) | 0.001 |

1. Achieving HBsAg < 10 IU/mL

| Variable | | Total number | Cases | PY | Annual incidences/1000 PY (95% CI) | Adjusted HR (95% CI) † | P |
| --- | --- | --- | --- | --- | --- | --- | --- |
| **HBeAg loss before year 3** | **No** | 431 | 11 | 5488.0 | 2.00 (1.11-3.62) | 1 | - |
| **HBeAg loss before year 3** | **Yes** | 301 | 49 | 3864.9 | 12.7 (9.58-16.8) | 6.25 (3.25-12.0) | < 0.001 |
| **HBeAg loss before year 5** | **No** | 298 | 6 | 4049.3 | 1.48 (0.67-3.30) | 1 | - |
| **HBeAg loss before year 5** | **Yes** | 360 | 45 | 4992.7 | 9.01 (6.73-12.1) | 5.91 (2.52-13.9) | < 0.001 |

**Supplementary Table 6.** Association between on-treatment HBeAg loss and HBsAg seroclearance or achieving HBsAg < 10 IU/mL among patients with baseline HBeAg positivity in the validation cohort (Landmark analysis)

†Adjusted for age and HBeAg loss


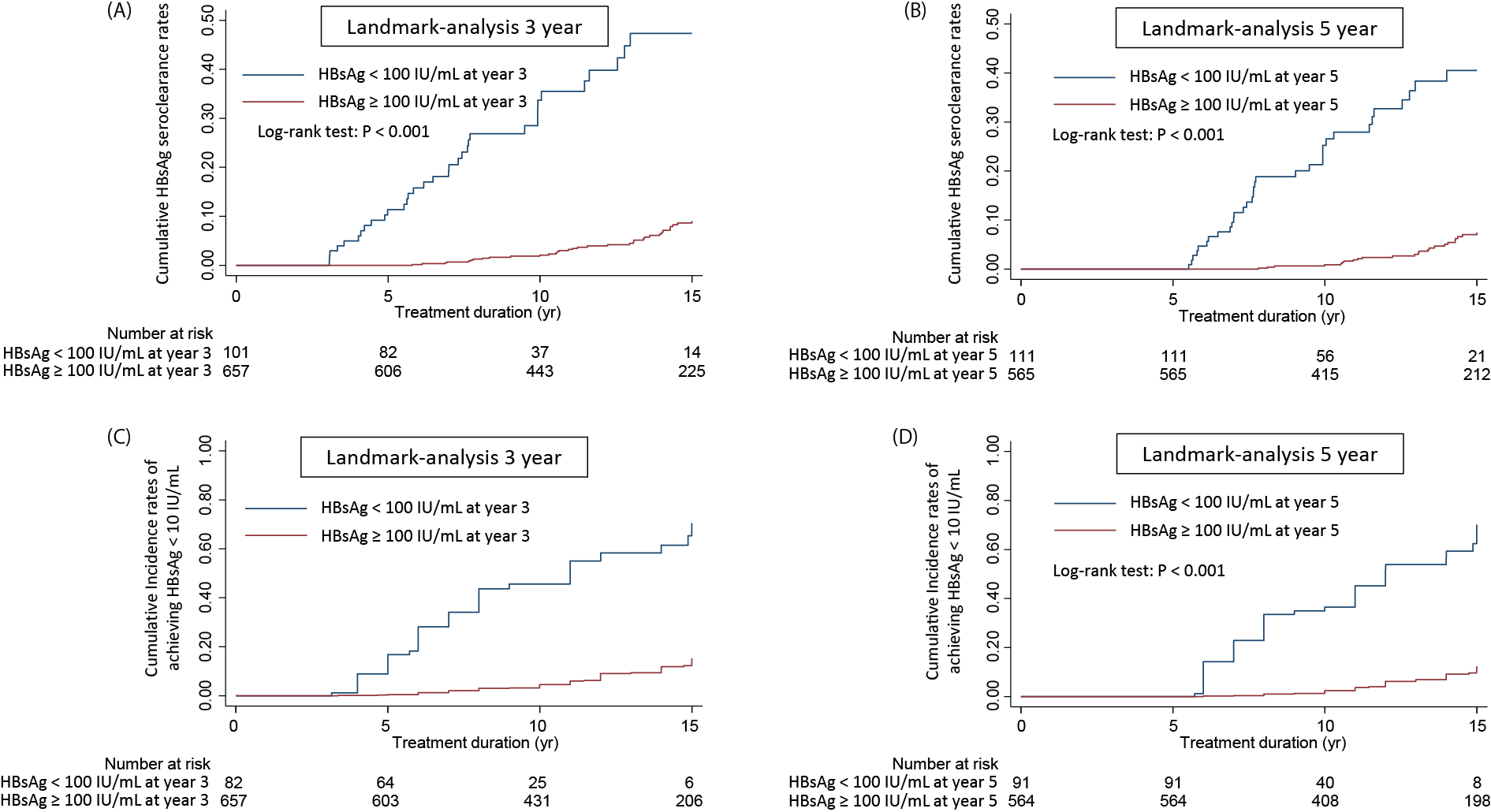


**Supplementary Figure 5.** Cumulative incidence rates of HBsAg seroclearance or achieving HBsAg < 10 IU/mL using the landmark-analysis among patients with baseline HBeAg negativity in the validation cohort (A) HBsAg seroclearance by HBsAg = 100 IU/mL at year 3. The log-rank test revealed a statistically significant difference in the HBsAg seroclearance between patients with HBsAg < 100 IU/mL at year 3 and without it (log-rank test: P < 0.001). (B) HBsAg seroclearance by HBsAg = 100 IU/mL at year 5. The log-rank test revealed a statistically significant difference in the HBsAg seroclearance between patients with HBsAg < 100 IU/mL at year 5 and without it (log-rank test: P < 0.001). (C) achieving HBsAg < 10 IU/mL by HBsAg = 100 IU/mL at year 3. The log-rank test revealed a statistically significant difference in the achievement HBsAg < 10 IU/mL between patients with HBsAg < 100 IU/mL at year 3 and without it (log-rank test: P < 0.001). (D) achieving HBsAg < 10 IU/mL by HBsAg = 100 IU/mL at year 5. The log-rank test revealed a statistically significant difference in the achievement of HBsAg < 10 IU/mL between patients with HBsAg < 100 IU/mL at year 5 and without it (log-rank test: P < 0.001).

(A)

| **Variable** | | **Total number** | **Cases** | **PY** | **Annual incidences/1000 PY (95% CI)** | **Adjusted HR (95% CI)** **†** | **P** |
| --- | --- | --- | --- | --- | --- | --- | --- |
| **HBsAg < 100 IU/mL at year 3** | **No** | 659 | 45 | 8440.5 | 5.33 (3.98-7.14) | 1 | - |
|  | **Yes** | 102 | 36 | 982.6 | 36.7 (26.4-50.8) | 7.97 (3.20-385.2) | < 0.001 |
| **HBsAg < 100 IU/mL at year 5** | **No** | 566 | 32 | 7731.1 | 4.14 (2.93-5.85) | 1 | - |
|  | **Yes** | 113 | 37 | 1254.0 | 29.5 (21.4-40.7) | 5.04 (1.87-13.5) | 0.001 |

(B)

| **Variable** | | **Total number** | **Cases** | **PY** | **Annual incidences/1000 PY (95% CI)** | **Adjusted HR (95% CI)** † | **P** |
| --- | --- | --- | --- | --- | --- | --- | --- |
| **HBsAg < 100 IU/mL at year 3** | **No** | 658 | 66 | 8248.0 | 8.00 (6.29-10.2) | 1 | - |
|  | **Yes** | 91 | 34 | 844.8 | 40.2 (28.8-56.3) | 5.87 (2.81-12.3) | < 0.001 |
| **HBsAg < 100 IU/mL at year 5** | **No** | 564 | 47 | 7595.0 | 6.19 (4.65-8.24) | 1 | - |
|  | **Yes** | 98 | 38 | 1001.7 | 37.9 (27.6-52.1) | 5.61 (2.57-12.3) | < 0.001 |

**Supplementary Table 7.** Association between on-treatment HBsAg level and HBsAg seroclearance or achieving HBsAg < 10 IU/mL among patients with baseline HBeAg negativity in the validation cohort (Landmark analysis)

†Adjusted for baseline and on-treatment HBsAg


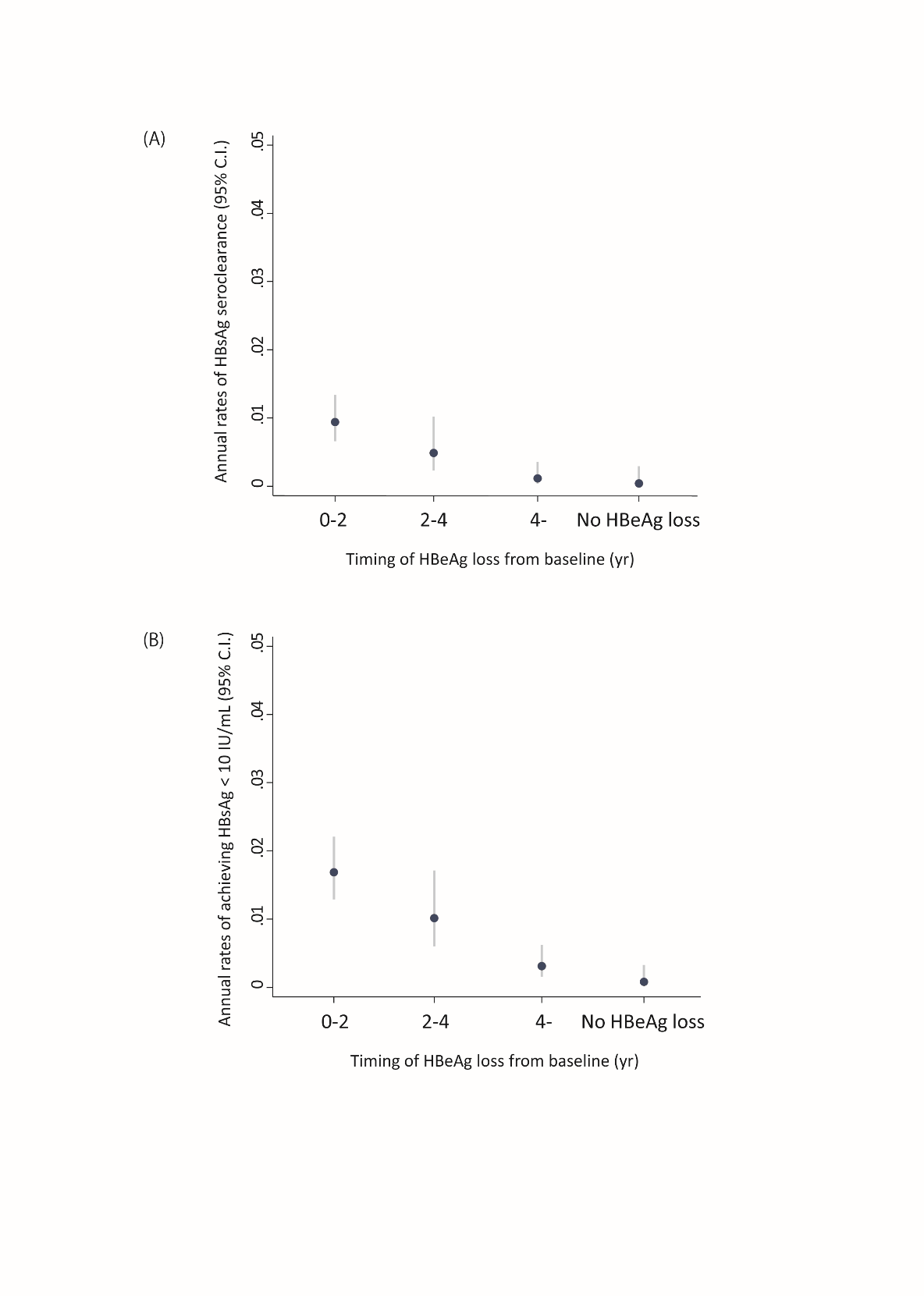


**Supplementary Figure 6.** Annual rates of HBsAg seroclearance or achieving HBsAg < 10 IU/mL among patients with baseline HBeAg positivity in the validation cohort by the timing of HBeAg loss (A) Annual rates of HBsAg seroclearance. The Mantel-Cox test revealed a statistically significant downtrend in the HBsAg seroclearance by the delayed timing of HBeAg loss (P for trend < 0.001). (B) Annual rates of achieving HBsAg < 10 IU/mL. The Mantel-Cox test revealed a statistically significant downtrend in the achievement of HBsAg < 10 IU/mL by the delayed timing of HBeAg loss (P for trend < 0.001).

| Derivation cohort with baseline HBeAg-positive | | | | |
| --- | --- | --- | --- | --- |
| Variable | Univariate OR (95% CI) | P | Multivariate-adjusted OR (95% CI) | P |
| **Age (y)** | 1.01 (0.99–1.03) | 0.418 |  |  |
| **Sex (male), no. (%)** | 1.12 (0.71–1.77) | 0.634 |  |  |
| **Cirrhosis, no. (%)** | 1.36 (0.80–2.33) | 0.261 |  |  |
| **HBV DNA (log copies/mL)** | 0.75 (0.61–0.92) | 0.005 |  |  |
| **HBsAg >250 IU/mL** | 1.48 (0.47–4.70) | 0.508 |  |  |
| **ALT level (IU/L)** | 0.99 (0.96–1.03) | 0.621 |  |  |
| **GGTP level (IU/L)** | 1.000 (0.997–1.003) | 0.979 |  |  |
| **Serum albumin (g/L)** | 0.88 (0.57–1.35) | 0.555 |  |  |
| **Platelet (105/mm^3^)** | 0.99 (0.95–1.03) | 0.568 |  |  |
| **History of IFN Tx** | 1.31 (0.72–2.37) | 0.378 |  |  |
| **First-line NUC**  **LAM**  **ETV**  **TDF or TAF** | 1  0.73 (0.44-1.20)  0.48 (0.05-4.77) | Ref.  0.215  0.528 |  |  |
| **Validation cohort with baseline HBeAg-positive** | | | | |
| Variable | Univariate HR (95% CI) | P | Multivariate-adjusted HR (95% CI)^#^ | P |
| **Age (y)** | 0.997 (0.985–1.009) | 0.626 |  |  |
| **Sex (male), no. (%)** | 1.12 (0.83–1.52) | 0.454 |  |  |
| **Cirrhosis, no. (%)** | 1.11 (0.77–1.61) | 0.563 |  |  |
| **HBV DNA (log IU/mL)** | 0.82 (0.74–0.90) | <0.001 | 0.84 (0.75–0.93) | 0.001 |
| **HBsAg >250 IU/mL** | 0.83 (0.46–1.49) | 0.525 |  |  |
| **ALT level (IU/L)** | 1.03 (1.02–1.04) | <0.001 | 1.03 (1.02–1.05) | <0.001 |
| **GGTP level (IU/L)** | 1.003 (1.001–1.005) | 0.001 |  |  |
| **Serum albumin (g/L)** | 0.92 (0.65–1.28) | 0.605 |  |  |
| **Platelet (105/mm^3^)** | 0.98 (0.96-1.00) | 0.083 |  |  |
| **History of IFN Tx** | 1.68 (1.25–2.26) | 0.001 |  |  |
| **First-line NUC**  **LAM**  **ETV**  **TDF or TAF** | 1  0.50 (0.37-0.68)  0.43 (0.28-0.67) | Ref.  <0.001  <0.001 | 1  0.61 (0.44-0.83)  0.55 (0.35-0.88) | Ref.  0.002  0.013 |

# Adjusted for HBV DNA, ALT, GGTP, history of IFN Tx and first-line NUC

**Supplementary Table 8. Factors associated with early HBeAg loss before year 3 among patients with baseline HBeAg positivity in both derivation and validation cohort (Logistic regression).**

(A)


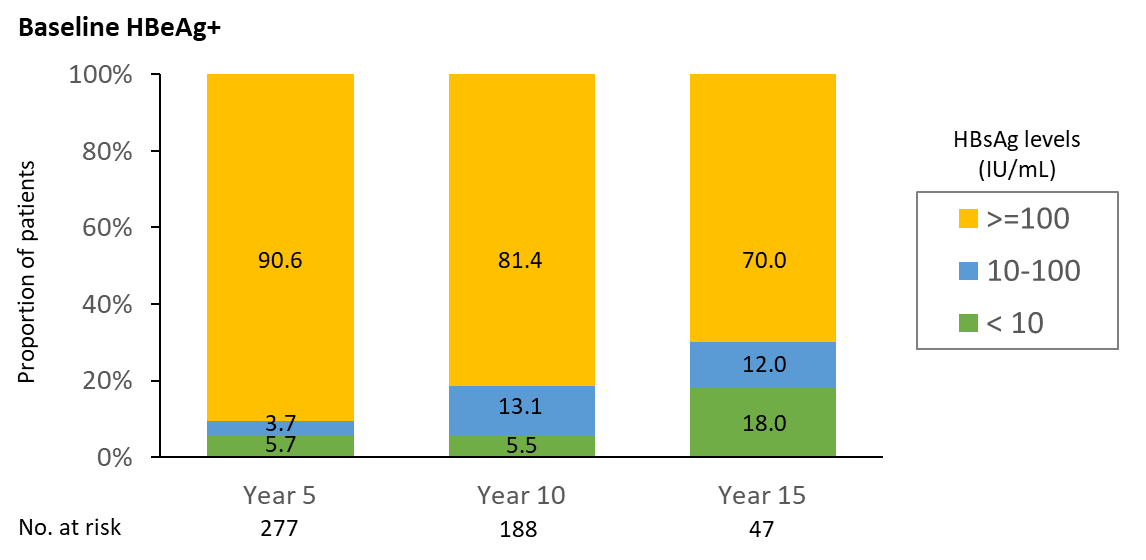


(B)


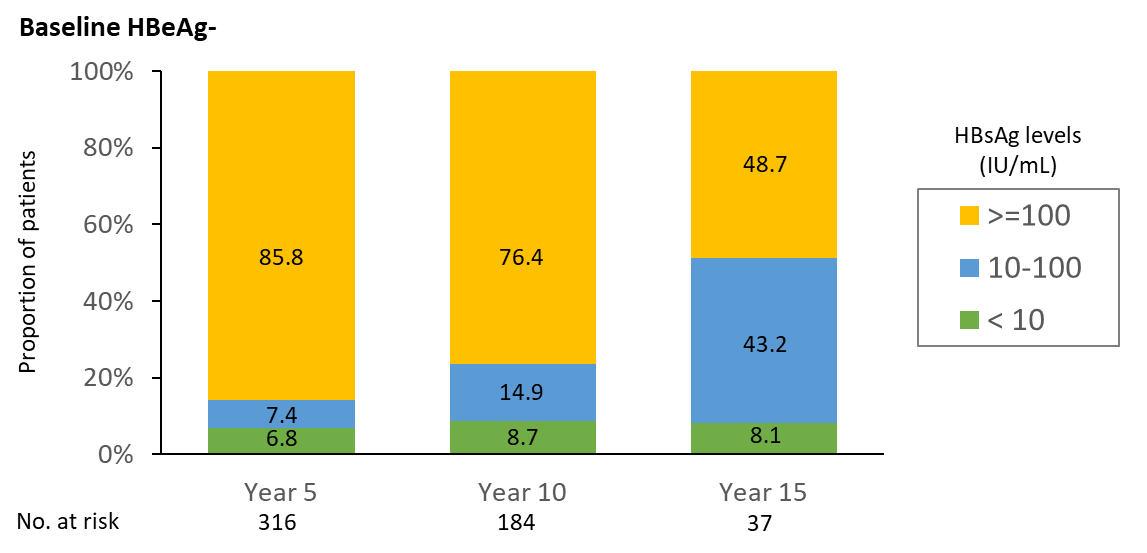


**Supplementary Figure 7.** The distribution of on-treatment HBsAg levels per 5 years by baseline HBeAg status in the derivation cohort. (A) Baseline HBeAg + (B) Baseline HBeAg -. Yellow boxes represent the proportion of patients with on-treatment HBsAg >= 100 IU/mL, blue boxes with 10 to 100 IU/mL, and green boxes with < 10 IU/mL at each time point.

(A)


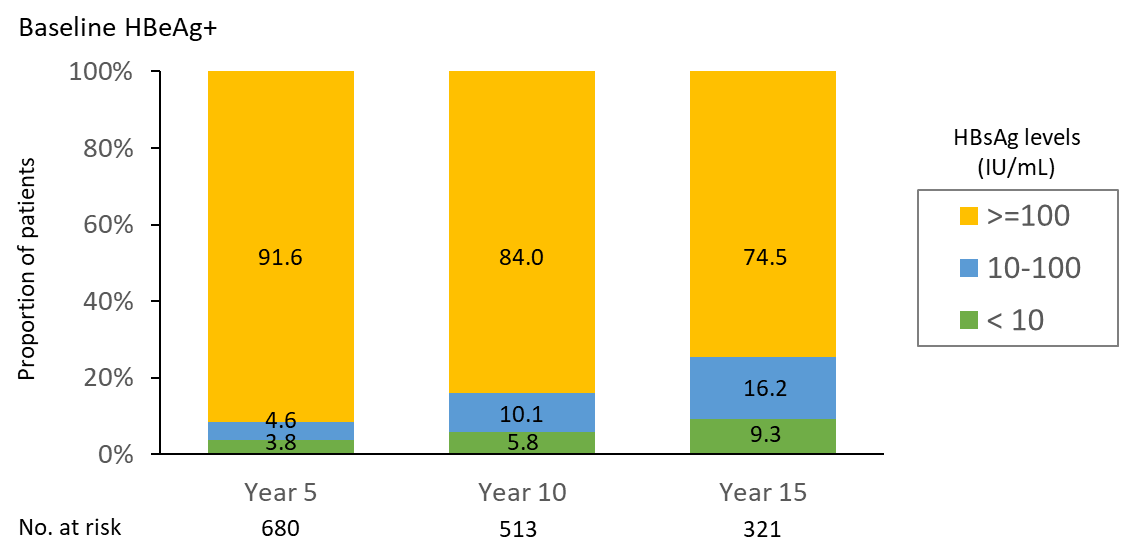


(B)


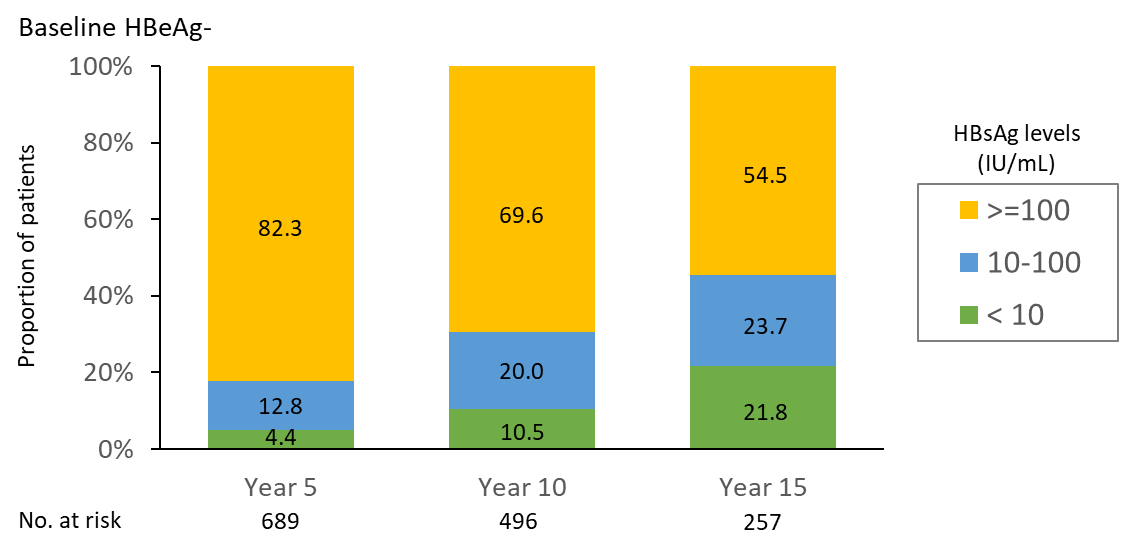


**Supplementary Figure 8.** The distribution of on-treatment HBsAg levels per 5 years by baseline HBeAg status in the validation cohort. (A) Baseline HBeAg + (B) Baseline HBeAg -. Yellow boxes represent the proportion of patients with on-treatment HBsAg >= 100 IU/mL, blue boxes with 10 to 100 IU/mL, and green boxes with < 10 IU/mL at each time point.
